# Study on the Molecular Basis of Huanglian Jiedu Decoction Against Atopic Dermatitis Integrating Chemistry, Biochemistry, and Metabolomics Strategies

**DOI:** 10.3389/fphar.2021.770524

**Published:** 2021-12-14

**Authors:** Jing Chen, Saizhen Chen, Jinguang Chen, Bixin Shen, Zhengli Jiang, Yubin Xu

**Affiliations:** ^1^ Department of Pharmacy, Taizhou Hospital of Zhejiang Province Affiliated to Wenzhou Medical University, Lin Hai, China; ^2^ Department of Pharmacy, Taizhou Central Hospital (Taizhou University Hospital), Taizhou, China; ^3^ Department of Dermatology, Taizhou Central Hospital (Taizhou University Hospital), Taizhou, China; ^4^ Department of Pharmaceutics, School of Pharmaceutical Sciences, Wenzhou Medical University, Wenzhou, China

**Keywords:** huanglian jiedu decoction, atopic dermatitis (AD), metabolomics, molecular basis, mechanism

## Abstract

Atopic dermatitis (AD) is a common chronic relapsing skin inflammation, which severely affect the quality of life of patients. Inhibiting itching and enhancing immunity to mitigate scratching are key elements in the fight against AD. Huanglian Jiedu decoction (HLJDD) has multiple pharmacological effects in the treatment of AD. However, the effective ingredients and underlying molecular mechanisms have not yet been fully explored. Thus, this study integrates chemistry, biochemistry, and metabolomics strategies to evaluate the active substance basis of HLJDD against AD. First, HLJDD was split to five fractions (CPF, 40AEF, 90AEF, PEF and WEF) and 72 chemical components were identified. NSD (Non-similarity degree) among the different fractions showed significant chemical differences (>81%). Interleukin IL-13, IL-17A, IL-3, IL-31, IL-33, IL4, IL-5, TSLP, IgE, and histamine in the serum, and IL-4Rα, JAK1, and HRH4 levels in skin, participating in inhibiting itching and regulating immunity signaling, were found to be restored to varying degrees in AD treating with HLJDD and its fractions, especially 40AEF and CPF. Untargeted metabolomics analysis demonstrated that forty metabolites were differential metabolites in plasma between the HLJDD-treated group and the AD group, involving in histidine metabolism, arginine biosynthesis, pyrimidine metabolism, and so on. Further, targeted metabolomics analysis revealed that eleven differential metabolites, associating with physiological and biochemical indices, were significant improved in the HLJDD and its fractions groups. In conclusion, HLJDD exhibited anti-AD effects by inhibiting itching and enhancing immunity, which in turn regulating the levels of relative metabolites, and CPF and 40AEF were considered the most important components of HLJDD.

## Introduction

Atopic dermatitis (AD), also known as atopic eczema, is a common chronic relapsing skin inflammation (Serrano et al., 2019). AD has a high incidence in developed countries, with a prevalence of up to 10–20% in Korea, Japan, Europe, and United States (Kim et al., 2016). As a result of rapid urbanization, the prevalence of AD has increased worldwide (Daya and Barnes, 2019). In China, a higher regional prevalence has been observed in coastal cities. Although AD is not uniformly lethal, it has a long disease duration and persistent symptoms, such as dryness, itching, and roughness, which severely affect the quality of life of patients, exerting a significant economic burden (Eckert et al., 2019; Olsson et al., 2020), especially in individuals with skin infections (Sandhu et al., 2019).

AD pathogenesis is a complex disorder that is considered to arise through a combination of genetic, immunological, environmental, skin barrier, and skin microbiota factors. Among these, skin barrier damage and immunologic dysfunction are considered critical factors (Bonamonte et al., 2019). Recent studies have shown that eosinophils and mast cells are the most important effector cells in AD, releasing inflammatory mediators, such as histamine, leukotrienes, and cytokines (IL-3, IL-4, IL-5, and IL-13), which ultimately leads to itching and skin lesions, which are characteristic of AD (Raap et al., 2006; Lam, 2010). A large number of CD4^+^ T-cell infiltrates were observed in AD. CD4^+^ T cells exhibit a variety of biological functions, including secretion of cytokines for immune regulation and effector function, which are divided into Th1 and Th2 subsets (Sur et al., 2016). Th2 cells secrete cytokines such as IL-4, IL-5, and IL-13, which are important for AD development, especially in the acute phase of AD. IL-4, IL-5, and IL-13 induce B cell activation to produce IgE, and there is a positive correlation between IgE and IL-4 levels. Mast cells were recruited by chemokines and activated by IgE or non-IgE factors. Finally, mast cells produce a range of inflammatory factors, which interact with nerve cells, T cells, eosinophils, and keratinocytes, and participate in the process of skin itch and inflammatory reactions in AD. In addition, IL-31 was not only strongly associated with pruritus in AD but also exhibited synergy with the H4 receptor, thus aggravating pruritus and skin lesions (Otsuka et al., 2011). Previous studies have indicated that IL-4Rα and JAK1 are key indices of inflammatory chronic itching and non-inflammatory chronic itching, respectively, with IL-4 promoting histamine release and worsening pruritus (Oetjen et al., 2017). Ultimately, skin barrier dysfunction and imbalanced immune function jointly contribute to the development of AD. Therefore, inhibiting itching and enhancing immunity to mitigate scratching are key elements in the fight against AD, which would improve the patient’s quality of life. A vast variety of treatments, including emollient, anti-inflammatory, and antimicrobial therapies, have been prescribed for AD. However, even though these treatments seem optimal in many patients, their long-term use carries the risk of severe systemic and cutaneous adverse effects. For these reasons, patients often seek alternative therapies (Blume-Peytavi et al., 2019). Therefore, a cure for AD requires a new treatment strategy to improve the efficacy and reduce the toxicity of short- and long-term therapeutic regimens.

Huanglian Jiedu decoction (HLJDD), a traditional Chinese medicine, is frequently prescribed due to its effectiveness against a variety of serious illnesses, including those of the skin (Chen et al., 2016). The formula is composed of four Chinese medicines: *Coptis chinensis* Franch.(Huanglian), *Scutellaria baicalensis* Georgi (huangqin), *Phellodendron amurense* Rupr.(huangbai), and *Gardenia jasminoides* J. Ellis (zhizi) at a ratio of 3:2:3:3 (Chen, et al., 2017). Modern pharmacological studies have confirmed that HLJDD has antipyretic, anti-inflammatory, antibacterial, antiulcer, and antitumor properties and promotes phagocytic function. With these properties, HLJDD has a curative effect in the treatment of AD (Qi et al., 2019). Clinical and experimental studies have confirmed that HLJDD is an effective drug for treating AD. However, the effective ingredients and underlying molecular mechanisms have not yet been fully explored (Kobayashi et al., 2004).

Traditional Chinese medicine (TCM) has been used to control and cure diseases for thousands of years. Formulas are a fundamental and unique part of TCM. TCM formulae are designed according to dialectical thinking, holistically and synergistically to maximize their therapeutic effectiveness. TCM formulae contains hundreds of chemical components, wherein each component has a different target, exhibiting extraordinary pharmacological activities (Zhang et al., 2017). In recent decades, decoction or ethanol extracts of Chinese drugs or TCM formulas have been split into different fractions to evaluate their main pharmacodynamic components (Pico-Hernández et al., 2020). However, many similar chemical components exist among the splitting fractions, resulting in a dispersion or loss of pharmacological activity. Hence, there is a need to establish a method with which to evaluate the non-similarity degrees of the splitting fractions.

At present, splitting fractions are characterized by HPLC to evaluate the non-similarity degrees among different fractions (Yuansheng et al., 2006). In our previous study, the chemical compositions of *Atractylodes macrocephala* Koidz. were split into five splitting fractions, and non-similarity degrees were evaluated using a variety of analytical methods (Li et al., 2014a). However, the characterization of these chemical constituents is often identified by HPLC techniques combining pure standards (Maimaiti et al., 2020). There are relatively few chemical constituents for characterization by HPLC, which could result in errors or an incomplete evaluation of non-similarity degrees. So far, there have been few reports on the characterization of chemical constituents of fractions of UPLC-Q-TOF/MS/MS, and almost no research on the evaluation of non-similarity degrees among different fractions. In recent years, LC-MS and UPLC-Q-TOF/MS/MS have been used to analyze and determine the chemical constituents of Chinese drugs, particularly UPLC-Q-TOF/MS/MS technology (Cao et al., 2015; Ma et al., 2016). Currently, UPLC-Q-TOF/MS/MS is widely used for the analysis of the multiple chemical constituents of Chinese drugs, and is highly accurate, highly efficient, and has a wide molecular mass range (Wang et al., 2015; Yang et al., 2015).

In the present study, the chemical constituents of HLJDD were split using various separation methods, analyzed, and identified by UPLC-Q-TOF/MS/MS technology combined with standard substances and literature. Meanwhile, a DNFB-induced AD model was established and used to explore the effects of HLJDD and its fractions. In addition, untargeted metabolomics analyses were performed to screen potential biomarkers of AD, and targeted metabolomic analyses were performed to elucidate and verify the therapeutic effects of HLJDD and its fractions.

## Materials and Methods

### Chemicals and Reagents

*Coptis chinensis Franch*.(Huanglian), *Scutellaria baicalensis* Georgi (huangqin), *Phellodendron amurense* Rupr.(huangbai), and *Gardenia jasminoides* J. Ellis (zhizi) were purchased from Hangzhou Mintai (Bozhou) Chinese Herbal Medicine Co. Ltd. (Zhejiang, China) and were certified by the standards elaborated in Chinese Pharmacopeia (2020 edition). Portions of the above four herbs were deposited in the Department of Pharmacy of Taizhou Central Hospital (Taizhou University Hospital), Taizhou, Zhejiang for future reference under reference number Xu1-4. 2,4-dinitrofluorobenzene (DNFB) was purchased from Shanghai McLean Biochemical Technology Co., Ltd. (Shanghai, China).

Methanol, acetonitrile, and formic acid (LC-MS grade) were purchased from CNW Technologies (Germany). Ethanol, petroleum ether, and ethyl acetate (analysis grade) were obtained from Tianjin Damao Chemical Reagent Co., Ltd. (China). The standard substances of baicalin, chrysin, ferulic acid, geniposide, obacunone, wogonin, wogonoside, auraptene, protopine, isochlorogenic acid A, chelerythrine, rutecarpine, palmatine, and berberine were purchased from the resource platform of the National Standard Material (China).

### Preparation of HLJDD and Splitting of the HLJDD Fractions

HLJDD was composed of *Coptis chinensis* Franch. 900 g, *Scutellaria baicalensis* Georgi 600 g, *Phellodendron amurense* Rupr. 600 g, and *Gardenia jasminoides* J. Ellis 900 g. The above four Chinese medicines were pulverized and decocted twice by refluxing with water (1:12 w/v) for 2 h, then filtered, and the filtrate was combined and concentrated in vacuum to 0.3 g crude drug per 1 ml and then stored in 4°C for the water decoction of HLJDD. According to the polarity differences of the chemical constituents of HLJDD, the water decoction of HLJDD was split into crude polysaccharide fraction (CPF), petroleum ether fraction (PEF), water eluted fraction (WEF), 40% ethanol eluted fraction (40AEF), and 90% ethanol eluted fraction (90AEF). The specific processes are described in the Supplementary Material S1. Meanwhile, the HPLC fingerprints of four small molecular components (PEF, WEF, 40AEF, and 90AEF) were established using a similarity evaluation system for chromatographic fingerprints of TCM (version 2012A). The chromatographic conditions are described in the Supplementary Material S1.

### UPLC-Q-TOF-MS/MS Analysis of HLJDD and its Fractions

The chemical constituents of HLJDD and its fractions were analyzed and identified by UPLC-Q-TOF/MS/MS technology combined with standard substances and the literature. Details of the chromatography, mass spectrometry conditions, and data processing are described in Supplementary Material S2.

### Non-Similarity Degree Evaluation of Chemical Fractions

Given that the overlapping property was small between CPF and other fractions, we only evaluated the NSD of four small molecular components (PEF, WEF, 40AEF, and 90AEF). NSD was evaluated and measured by qualitative analysis and quantitative analysis using HPLC data and UPLC-Q-TOF-MS/MS data.

Principal component analysis (PCA) and hierarchical clustering analysis (HCA) were used to evaluate the NSD of the four fractions. Multivariate analysis was performed using a combination of PCA and the SIMCA-P software (version 14.0). HCA was performed using the OmicShare tools in RStudio.

Based on a previous study (Li et al., 2014b), the standard degree was defined as the overlapping property or similarity degree, and NSD was defined as the un-overlapping property or non-similarity degree among the four fractions. The sum of the similarity degree and NSD was equal to 1. Furthermore, NSD was introduced to measure the un-overlapping property of four fractions by peak areas of crude drug, angle cosine analysis, and correlation coefficient analysis. NSD among different fractions was calculated using the cross-analysis method of peak areas of crude drug, angle cosine analysis method (Wang et al., 2002), and correlation coefficient analysis method (Guo et al., 2014). The specific algorithm for the three methods is described in Supplementary Material S3.

### AD Model Establishment and Treatment

Male C57BL/6 mice (4–6 weeks), weighing 20 ± 2 g, were purchased from Shrek Jinda Laboratory Animals Co., Ltd. (Hunan, China, Laboratory Animal License #SCXK 2016-0010). The animals were kept under SPF laboratory conditions and provided with a standard laboratory diet and filtered tap water. All animal experimental procedures were approved by the Bioethics Committee of Shanghai Tenth People’s Hospital (ID no. SHDSYY-2020-9001). All experimental animals were acclimated for 1 week before the experiment.

In the present study, reference-dose HLJDD in mice was selected as the clinically equivalent dose of HLJDD in humans (according to the body surface area exchange algorithm of human and mice: HLJDD clinical dose: 30 g/day, adult weight 70 kg, the dose of mice was: 30 g/70 kg × 9.1 ≈ 3.9 g/kg). With this as a reference dose, we have previously shown the effects of different dosages of HLJDD (3.2 g/kg, 6.4 g/kg, and 12.8 g/kg) on the AD model. After the dose-effect experiment, 12.8 g/kg was found to be the effective dose (Supplementary Material S4). Therefore, the 12.8 g/kg water decoction of HLJDD and the five fractions with the corresponding extracts (CPF: 921.6 mg/kg, 40AEF: 458.24 mg/kg, 90AEF: 460.8 mg/kg, WEF: 1.0086 g/kg, PEF: 1.38 mg/kg) were used to evaluate the mechanism and substance basis of Huanglian Jiedu decoction against AD. Then, C57BL/6 mice were randomly divided into 8 groups (8 mice per group): normal control group, AD model group, HLJDD group (12.8 g/kg), CPF group, 40AEF group, 90AEF group, WEF group, and PEF group. To prepare the 2,4-dinitro-1-fluorobenzene (DNFB) solution, DNFB was dissolved in acetone/olive oil (4:1) and applied to mouse skin. The procedure was performed as previously reported, with some modifications (Chen et al, 2020a). Briefly, an electric shaver was used to remove the hair from the abdominal and back skin of mice on the first day (the area of hair removal was about 2 × 2 cm^2^). Then, 200 μl of 1% DNFB was used to sensitize the abdominal skin on days 1, 4, and 7, once a day, for a total of three times. The mice were challenged with 200 μl of 0.5% DNFB on the back skin on days 14, 17, 19, 22, 24, 27, and 29 in the model and HLJDD groups, once a day. Mice from the control group were smeared with an equal volume of DNFB matrix solution at the same time points. After being challenged, the mice from the HLJDD groups and its fractions groups were intragastrically administrated with HLJDD (12.8 g/kg) and its fractions (CPF: 921.6 mg/kg, 40AEF: 458.24 mg/kg, 90AEF: 460.8 mg/kg, WEF: 1.0086 g/kg, PEF: 1.38 mg/kg) once a day for 16 days, respectively, while the mice from normal control group and AD model group were administrated with an equal volume of saline. After the last administration, each group of mice was anesthetized. Blood samples were collected from the ophthalmic venous plexus, from which serum was separated and stored at ‒80°C for future research. Finally, the mice were sacrificed, and the dorsal skin tissue samples were harvested and stored at ‒80°C for further analysis.

### Evaluation of Back Skin Lesions

The AD-like symptoms of skin induced by DNFB were scored upon completion of drug treatment. The back lesions were evaluated based on clinical presentation criteria (Choi et al., 2018): erythema, edema/papules, epidermal stripping/scratches, and scales (dry), which were categorized into four grades: no lesion, light, medium, and severe (recorded as 0, 1, 2, and 3, respectively). Ear thickness was measured using a Vernier caliper, and the difference between the weight of the left and right ears was calculated as the degree of swelling. The swelling rate of the ear was calculated as follows (Li et al., 2020): [(the degree of swelling of ear in AD model group ‒ the degree of swelling of ear after treatment)/the degree of ear swelling in the AD model group] × 100%.

### H&E Staining

The dorsal skin was embedded in paraffin and sliced into 5-μm sections. The pathological sections were stained with hematoxylin and eosin (H&E), and the sections were examined under an optical microscope.

### Index Measurement

The serum levels of IgE and histamine were determined using the appropriate ELISA kits according to the manufacturer’s instructions. The serum levels of IL-13, IL-17A, IL-3, IL-31, IL-33, IL4, IL-5, and TSLP in the serum were measured using Luminex technology (R&D Systems, Abingdon, United Kingdom) on a Luminex 200 instrument.

### Real-Time PCR Analysis

RT-PCR was used to determine the expression levels of JAK1, HRH4, and IL-4Rα mRNAs. Total RNA was isolated and extracted from the dorsal skin tissues using TRIzol^®^ reagent (Invitrogen, Thermo Fisher Scientific, Inc.). Reverse transcription was performed to synthesize cDNA using the HiScript II Q RT SuperMix for qPCR (+gDNA wiper) (R223-01; Vazyme, Inc.). RT-PCR analysis was performed using 2× SYBR Green PCR Master Mix (A4004M; Lifeint) on a CFX Connect™ PCR instrument (Bio-Rad Laboratories, Shanghai, China) according to the manufacturer’s protocol. The primer sequences were as follows: JAK1 specific primer (forward: 5′-GGC​GTT​CTG​TGC​TAA​AAT​GA-3′, reverse: 5′-AGG​GCG​AAG​AGG​TTG​TGA​C-3′), IL-4Rα specific primer (forward: 5′-GGA​GGA​GGA​AGA​AGA​TGA​GAT​AG-3′, reverse: 5′-CCA​ACA​AGT​CGG​AAA​ACA​GG-3′), HRH4 specific primer (forward: 5′-TGA​CTT​CCT​CGT​GGG​TTT​G-3′, reverse: 5′-ATT​GTA​GAC​AGA​TGC​GGT​GC-3′), and β-actin specific primer (forward: 5′-AGG​GAA​ATC​GTG​CGT​GAC-3′, reverse: 5′-CAT​ACC​CAA​GAA​GGA​AGG​CT-3′). The expression levels of the target mRNAs were calculated based on normalization to β-actin expression.

### Western Blot Analysis

Western blotting was performed to evaluate the protein expression of IL-4Rα, JAK1, p-JAK1, and HRH4. Briefly, total protein from dorsal skin tissue was extracted and quantified using the BCA protein quantitative assay. Protein sample buffer was added and heated in a boiling water bath. The denatured protein was separated on a 12% polyacrylamide gel and transferred to a polyvinylidene fluoride membrane. The blot was blocked in 5% skimmed milk at room temperature for 2 h, followed by co-incubation with the appropriate proportions of primary and secondary antibodies. The number of target bands was detected using ECL chemiluminescence. The antibodies used for western blotting were anti-IL-4Rα (#DF8567; Affinity), anti-JAK1 (#66466-1-ap; Proteintech), anti-p-JAK1 (#AF 2012; Affinity), and anti-HRH4 (#OM209489; Omnimabs).

### Untargeted Metabolomics

To explore the changes in the metabolomic characteristics of AD, the concentrations of different serum metabolites in AD and HLJDD-treated mice were confirmed by a UPLC-Q-TOF/MS/MS-based metabolomic approach. Details of chromatography, mass spectrometry conditions, and data processing are provided in Supplementary Material S5.

### Targeted Metabolomics

To screen for discriminatory metabolites, Pearson’s correlation analysis was performed to analyze the correlation between macroscopic indexes and metabolites. Metabolites significantly associated with macroscopic indices were used to further evaluate the mechanism of HLJDD fractions. Meanwhile, considering the accuracy of metabolites and the diversity detected in each sample, we performed a targeted quantification of 14 endogenous metabolites (targeted metabolomics). The standard substances (prostaglandin D1, phosphocreatinine, n-methyl-d-aspartic acid, n-hydroxymethylnorcotinine, malic acid, lysopc (24:1 (15z)), linolelaidic acid, l-alloisoleucine, l-alanine, humulinone, glutaric acid, gamma-linolenic acid, dihydro-o-methylsterigmatocystin, deoxyuridine, d-phenyllactic acid, carnosine, biliverdin, alanyl-glutamine, 4-imidazolone-5-propionic acid, 3,7-dimethylpurine-2,6-dione, 2-hydroxy-2-methylbutanedioic acid, 1h-pyrimidine-2,4-dione, and (z)-tetracos-15-enoic acid) were dissolved in a diluent (methanol/acetonitrile = 1:1) for further study.

Details of sample processing, chromatography, mass spectrometry conditions, and data processing are described in Supplementary Material S6, and the MRM parameters of standard substances are given in Supplementary Table S1.

Furthermore, receiver operating characteristic (ROC) curve analysis and area calculation curve (AUC) under ROC were performed to evaluate the change in the diagnostic ability of potential biomarkers.

### Statistical Analysis

All values are presented as the mean ± standard deviation (SD). The significance of differences among the groups was determined by one-way analysis of variance (ANOVA) using the statistical package for the social science program (SPSS 19.0, Chicago, IL, United States). Statistical significance was set at *p* < 0.05.

## Results and Discussion

### Splitting HLJDD Fractions and Establishment of HPLC Fingerprints

HLJDD was split into five fractions: CPF (yield, 7.200 ± 0.731%, *n* = 5), PEF (yield, 0.0108 ± 0.002%, *n* = 5), 40AEF (yield, 3.580 ± 0.179%, *n* = 5), 90AEF (yield, 3.60 ± 0.752%, *n* = 5), and WEF (yield, 7.880 ± 0.928%, *n* = 5). The HPLC fingerprints of four fractions (WEF, 40AEF, 90AEF, and PEF) were successfully established, and the similarity of eight batches of samples was above 0.9, suggesting that the stability of the process was relatively good (Supplementary Figures S1–S4).

### Qualitative Analysis by UPLC-Q-TOF/MS

According to the analysis of the mass fragment information of each chemical component in the Massbank database, 72 chemical components were identified and compared with the literature data. Among these major constituents, 15 alkaloids, 22 flavonoids, 6 iridoids, 18 organic acids and amino acids, and 11 other constituents were identified in this experiment (Table 1).

**TABLE 1:** UPLC-Q-TOF/MS identification of the constituents in HLJDD.

NO.	t_R_ (min)		Positive ion MS	Negative ion MS	Formula	Identifcation	Positive ion MS/MS	Negative ion MS/MS	Relegation
1	0.58	[M-H]^-^	-	191.0558	C_7_H_12_O_6_	Quinic acid	-	191.0554, 127.0395, 85.0289	Organic acid
2	0.63	[M-H]^-^	-	133.0142	C_4_H_6_O_5_	Malic acid	-	115.0030, 71.0134	Amino acid
3	0.63	[M + H]^+^	175.1187	-	C_6_H_14_N_4_O_2_	Arginine	70.0649	-	Amino acid
4	0.65	[M + H]^+^	116.0699	-	C_5_H_9_NO_2_	Proline	70.0648	-	Amino acid
5	0.79	[M + H]^+^	182.081	-	C_9_H_11_NO_3_	Tyrosine	96.0437	-	Amino acid
6	0.81	[M-H]^-^	-	191.0201	C_6_H_8_O_7_	Citric acid	-	173.0077, 111.0079. 87.0080	Organic acid
7	0.86	[M + H]^+^	268.1041	-	C_10_H_13_N_5_O_4_	Adenosine	136.0310, 119.0341	-	Nitrogen glycosides
8	1.02	[M + H]^+^	132.1011	-	C_6_H_13_NO_2_	Isoleucine	86.0960, 69.0688	-	Amino acid
9	1.51	[M + H]^+^	139.0384	-	C_7_H_6_O_3_	3.4.Dihydroxybenzaldehyde	136.0181, 119.0160, 108.0228. 81.0360	-	Organic acid
10	1.53	[M + H]^+^	166.0859	-	C_9_H_11_NO_2_	Phenylalanine	120.0801, 103.0534. 91.0538. 77.0380	-	Amino acid
11	1.6	[M + H]^+^	127.0383	-	C_6_H_6_O_3_	Maltol	103.0548. 77.0385	-	Saccharides
12	1.64	[M-H]^-^	-	197.0458	C_9_H_10_O_5_	danshensu	-	179.0351, 135.0453	Organic acid
13	1.71	[M-H]^-^	-	391.1240	C_16_H_24_O_11_	Shanzhiside	-	229.0714, 185.0821, 167.0714	Iridoid
14	1.78	[M-H]^-^	-	373.1135	C_16_H_22_O_10_	Geniposidic acid	-	211.0611, 149.0609, 123.0446	Iridoid
15	2.21	[M + H]^+^	208.0974	-	C_11_H_13_NO_3_	Hydrastinine	190.086, 175.0612, 147.0432	-	Alkaloids
16	2.28	[M + H]^+^	192.0419	-	C_10_H_8_O_4_	7,8-Dihydroxy-4-methylcoumarin	175.0386, 147.0428, 119.0482, 91.0543	-	Coumarin
17	2.98	[M + Na]^+^	427.1201	-	C_17_H_24_O_11_	Gardenoside	265.0683, 247.0575, 233.6419, 215.0310	-	Iridoid
18	2.33	[M + H]^+^	188.0704	-	C_11_H_9_NO_2_	3-Indoleacrylic acid	143.0727, 118.0647	-	Organic acid
19	2.33	[M + H]^+^	205.0969	-	C_11_H_12_N_2_O_2_	Tryptophan	188.0694	-	Amino acid
20	2.41	[M + H]^+^	272.1279	-	C_16_H_17_NO_3_	Higenamine	255.1015, 161.0590, 107.0488	-	Alkaloids
21	2.67	[M-H]^-^	-	153.0194	C_7_H_6_O_4_	Gentisic acid	-	108.0229. 81.0348. 53.0394	Organic acid
22	2.89	[M-H]^-^	-	369.1516	C_16_H_26_O_8_	Jasminoside B	-	339.1420	Monoterpenoids
23	2.9	[M + H]^+^	355.102	353.0874	C_16_H_18_O_9_	Chlorogenic acid	163.0380, 145.0279, 135.0437	353.0874, 91.0561, 179.0358, 35.0448	Organic acid
24	3.09	[M + Na]^+^	395.1305	-	C_17_H_24_O_9_	Eleutheroside B	364.1139, 232.0708	-	-
25	3.12	[M-H]^-^	-	353.0873	C_16_H_18_O_9_	4-Ocaffeoylquinic acid	-	353.0877, 173.0454, 135.0446	Organic acid
26	3.28	[M + HCOOH-H]^-^	-	595.1876	C_23_H_34_O_15_	Genipin gentiobioside	-	517.1567, 323.0988, 225.0769, 207.0663	Iridoid
27	3.29	[M + Na]^+^	573.1782	-	C_23_H_34_O_15_	Genipin-1-gentiobioside	541.1509, 511.1411, 365.1031, 347.0937, 305.0835	-	Iridoid
28	3.38	[M + Na]^+^	411.1264	-	C_17_H_24_O_10_	Geniposide^a^	379.0960, 349.0902, 249.0728, 231.0618,217.0467, 203.0527, 185.0415, 147.0411	-	Iridoid
29	3.76	[M + H]^+^	169.0755	-	C_11_H_8_N_2_	Norharmane	115.0539	-	-
30	3.77	[M-H]^-^	-	341.1622	C_20_H_24_NO_4_	Phellodendrine	-	-	Alkaloids
31	4.8	[M + H]^+^	195.0657	-	C_10_H_10_O_4_	Ferulic Acid^a^	145.0279, 117.0326. 89.0379	-	Organic acid
32	5.14	[M + NH_4_]^+^	207.0646	-	C_11_H_12_O_5_	3,5-Dimethoxy-4-hydroxycinnamic acid	192.0410, 147.0445, 119.0498	-	Organic acid
33	5.18	[M + H]^+^/[M-H]^-^	611.1601	609.1458	C_27_H_30_O_16_	Rutin	303.0491	-	Flavonoid
34	5.18	[M + H]^+^	303.0494	-	C_15_H_10_O_7_	Quercetin	-	285.0409, 257.0419, 229.0484	Flavonoid
35	4.8	[M + H]^+^	322.1069	-	C_19_H_15_NO_4_	Berberrubine	307.0814, 294.1114, 279.0865	-	Alkaloids
36	5.54	[M + H]^+^	509.1281	-	C_23_H_24_O_13_	Syringetin 3-glucoside	347.0758, 332.0528	-	Phenylethanoid glycoside
37	5.76	[M + H]^+^	352.1174	-	C_20_H_17_NO_5_	8-O-berberine	337.0929, 308.0900	-	Alkaloids
38	5.78	[M-H]^-^	-	515.1188	C_25_H_24_O_12_	Isochlorogenic acid A^a^	-	-	Organic acid
39	5.83	[M + H]^+^	356.1846	-	C_21_H_25_NO_4_	Tetrahydropalmatine	192.1004, 177.0770, 165.0915	-	Alkaloids
40	5.89	[M-H]^-^	-	623.1974	C_29_H_36_O_15_	Methyl hesperidin	-	461.1673, 415.1035, 295.0622, 161.0248	Flavonoid
41	5.99	[M + H]^+^	354.1337	-	C_20_H_19_NO_5_	Protopine^a^	-	-	Alkaloids
42	6.14	[M]^+^	336.1221	-	C_20_H_18_NO_4_	Epiberberine	320.0892, 292.0942	-	Alkaloids
43	6.25	[M]^+^	338.1393	-	C_20_H_20_NO_4_	Dihydroberberine	322.1048, 294.1098	-	Alkaloids
44	6.28	[M]^+^	461.1071	-	C_22_H_21_O_11_	Oroxylin A 7-O-glucuronide	285.0761, 270.0530	-	Flavonoid
45	6.28	[M + H]^+^	523.1435		C_24_H_26_O_13_	Iridin	361.0900, 331.0438	-	Flavonoid
46	6.35	[M]^+^	320.0913	-	C_19_H_14_NO_4_	Coptisine	292.0592	-	Alkaloids
47	6.53	[M]^+^	352.1197	-	C_21_H_22_NO_4_	Palmatine^a^	336.1232, 308.1289	-	Alkaloids
48	6.71	[M]^+^	336.1222	-	C_20_H_18_NO_4_	Berberine^a^	320.0876, 306.0749, 304.0956, 292.0942, 278.0787	-	Alkaloids
49	6.8	[M + H]^+^	463.0861	-	C_21_H_18_O_12_	Scutellarin	287.0548, 269.0444, 241.0488	-	Flavonoid
50	6.88	[M + H]^+^	417.1173	-	C_21_H_20_O_9_	Chrysin 8-C-glucopyranoside	399.1088, 297.0758, 267.0653	-	Flavonoid
51	7.08	[M + H]^+^	273.0748	-	C_15_H_12_O_5_	Dihydronorwogonin	169.0124	-	Flavonoid
52	7.17	[M + H]^+^	447.0917	-	C_21_H_19_O_11_	Baicalin^a^	271.0581, 169.0123	-	Flavonoid
53	7.22	[M + H]^+^	593.1871	-	C_28_H_32_O_14_	Linarin	447.1269, 285.0755	-	Flavonoid
54	7.42	[M-H]^-^	-	459.0923	C_22_H_20_O_11_	Wogonoside^a^		459.0907, 268.0370, 239.0349	Flavonoid
55	7.46	[M-H]^-^	-	429.0816	C_21_H_18_O_10_	Chrysin-7-O-β-d-glucuronide	-	253.0508, 175.0249, 113.0255	Flavonoid
56	7.6	[M-H]^-^	-	475.0874	C_22_H_20_O_12_	Hydroxyl oroxylin A-7-O-β-d-glucutonide		299.0555, 284.0316, 255.0298,175.0246, 113.0241	Flavonoid
57	7.75	[M-H]^-^	331.0806	-	C_17_H_14_O_7_	5, 7, 4' -trihydroxy-3 ′, 5' -dimethoxy flavone	-	316.0588, 270.0507	Flavonoid
58	8.02	[M-H]^-^	-	299.0555	C_16_H_12_O_6_	Hispidulin		284.0342, 269.0359, 239.0371, 136.9880	Flavonoid
59	8.26	[M + H]^+^	260.0906	-	C_14_H_13_NO_4_	Skimmianine	245.0663, 227.0557	-	Alkaloids
60	8.49	[M-H]^-^	-	975.3713	C_44_H_64_O_24_	Crocin	-	651.2661, 327.1600	Flavonoid
61	9.2	[M-H]^-^	-	299.0557	C_16_H_12_O_6_	4 -Hydroxywogonina	-	284.0342, 239.0372,136.9880	Flavonoid
62	9.28	[M]^+^	470.1962	-	C_26_H_30_O_8_	Obaculactone	425.1936, 409.2004	-	-
63	9.66	[M + H]^+^	375.1045	-	C_19_H_18_O_8_	Casticin	375.0572	-	-
64	9.72	[M + H]^+^	285.0748	283.0612	C_16_H_12_O_5_	Wogonin^a^	270.0502	283.0608, 163.0027	Flavonoid
65	9.81	[M + H]^+^	255.0638	253.0506	C_15_H_10_O_4_	Chrysin^a^	-	209.0616 183.0472, 143.0509, 107.0143	Flavonoid
66	9.88	[M + H]^+^	315.0852	-	C_17_H_14_O_6_	3,7-Dihydroxy-3′,4′-dimethoxyflavone	300.0613, 285.0372, 257.0426	-	-
67	10.28	[M-H]^-^	-	343.0818	C_18_H_16_O_7_	5, 7-dihydroxy-2 ′,6 ′, 8-trimethoxylflavone	-	313.0351, 285.0405, 269.0402	Flavonoid
68	10.39	[M + H]^+^	286.1431	-	C_17_H_19_NO_3_	Piperine	-	-	Alkaloids
69	10.5	[M + H]^+^	455.2051	-	C_26_H_30_O_7_	Obacunone^a^	437.1929, 409.2004	-	Flavonoid
70	10.32	[M + H]^+^	288.1585	-	C_18_H_13_N_3_O	Rutecarpine^a^	-	-	Alkaloids
71	12.49	[M + H]^+^	299.1604	-	C_19_H_22_O_3_	Auraptene^a^	-	-	Flavonoid
72	18.82	[M + H]^+^	397.3814	-	C_29_H_50_O	β-Sitosterol	147.123	-	Sterols

a Indicates comparison with reference substance.

#### Alkaloids

*Coptis chinensis* Franch. and *Phellodendron amurense* Rupr. were found to be the main drugs of HLJDD, and contained large quantities of alkaloids, including coptisine, palmatine, and berberine. These ingredients of HLJDD exhibit a wide range of pharmacological effects and biological activities (Chen et al., 2020a). A total of 15 alkaloid chemical components (compound 15, 20, 30, 35, 37, 39, 41-43, 46-48, 59, 66, and 70) were identified in this study: hydrastinine, higenamine, phellodendrine, berberrubine, 8-O-berberine, tetrahydropalmatine, protopine, epiberberine, dihydroberberine, coptisine, palmatine, berberine, skimmianine, piperine, and rutecarpine. Berberine was used an example to evaluate the possible cleavage pathway of alkaloids. Compound 48 was determined as berberine using pure standard data and combining the fragmentation rules with data reported in the literature. First, berberine showed fragment ions at *m/z* 336.1222 [M]^+^, 320.0876 [M-CH_4_]^+^, 306.0749 [M-CH_4_-CH_2_]^+^, 292.0942 [M-CH_4_-CO]^+^, and 278.0787 [MCH_4_-CO-CH_2_]^+^. The fragmentation at *m/z* 320.0876 [M-CH_4_]^+^ was acquired at *m/z* 336.1222 [M]^+^ through the loss of a molecule of CH_3_ and a rearrangement reaction. Further, this fragmentation lost the methyl group at the end site to form fragment ions at *m/z* 306.0749 [M-CH_4_-CH_2_]^+^; on the other hand, this fragmentation also resulted in the formation fragment ions at *m/z* 292.0942 [M-CH_4_-CO]^+^ by the cracking of the side benzene ring and the loss of a molecule of CO, and continued to lose of the side ring-CH_2_ fragment and formed fragment ions at *m/z* 278.0787 [MCH_4_-CO-CH_2_]^+^. Combined with standard substance analysis and the literature (Zuo et al., 2014) this was inferred as berberine (Supplementary Figure S5).

#### Flavonoids

Flavonoids are the main active components in the form of either sugar glucoside or free molecules in natural plants. They possess many bio-activities, including lipid-lowering (Yao et al., 2014), antitumor (Wang et al., 2020), anti-oxidation (Wang et al., 2021), and anti-bacterial (Rasouli et al., 2019). Scutellariae Radix in HLJDT contains a large amount of flavonoids, such as quercetin, rutin, baicalin, wogonin, and chrysin (Yang et al., 2020). A total of 22 flavonoids (compound 33-34, 40, 44-45, 49-58, 60-61, 64-65, 67, 69, and 71) were identified in this study: rutin, quercetin, methyl hesperidin, oroxylin A 7-O-glucuronide, iridin, scutellarin, chrysin 8-C-glucopyranoside, dihydronorwogonin, baicalin, linarin, wogonoside, chrysin-7-O-β-d-glucuronide, Hydroxyl oroxylin A-7-O-β-d-glucutonide, 5,7,4′-trihydroxy-3′, 5′-dimethoxy, hispidulin, crocin, 4-hydroxywogonina, wogonin, chrysin, 5, 7-dihydroxy-2′,6′, 8-trimethoxylflavone, obacunone, and auraptene. Of these, baicalin, wogonoside, wogonin, chrysin, obacunone, and auraptene were identified using pure standards. Flavonoids often exist in the form of flavonoid glycosides. Therefore, the fragmentation regularity of flavonoids mainly includes the breakage of glycosidic bonds and flavonoid aglycon (Ferreres et al., 2004). For example, in the positive ion mode, baicalin (compound 19), excimer ions broke the glycosidic bond and formed fragment ions *at m/z* 271.0581 [M + H-GlucA]^+^. Furthermore, the flavonoid aglycon was opened and cleaved to *m/z* 169.0123 [M + H-GlucA-C_7_H_2_O]^+^. The mass spectrometry cleavage pathway is illustrated in Supplementary Figure S6.

#### Iridoids

Iridoids mainly come from gardeniae fructus and have hypoglycemic, anti-inflammatory, and anti-tumor effects (Hung et al., 2010; Koo et al., 2004; Xu and Hao, 2004). A total of six iridoids (compounds 13, 14, 17, 26, 27, and 28) were identified in this study. These were shanzhiside, geniposidic acid, gardenoside, genipin gentiobioside, genipin-1-gentiobioside, and geniposide by combining the fragmentation rules with data from the literature. Geniposide was identified using a standard. For example, the mass spectrometry cleavage pathway of geniposide (compound 28) is shown in Supplementary Figure S7. The molecular ion peak of *m/z* 411.1264 [M + Na]^+^ was obtained in positive mode, and hydrogen on the double bond appeared lively due to the influence of oxygen atoms, combining with the oxygen on the ester bond. After fracturing, the mother nucleus lost a molecule of CH_3_OH to form a fragment ion at *m/z* 379.0960, which continued to lose glucose or formaldehyde molecules to form fragment ions at *m/z* 217.0467 or 349.0902. The glycosidic bond of geniposide has two cleavage pathways. On the one hand, active hydrogen on the mother nucleus transferred and formed fragment ions at *m/z* 231.0618 and complete glucose fragment ions at *m/z* 203.0527. On the other hand, the active hydrogen on the glucose transferred and formed fragment ions at *m/z* 249.0728 and complete glucose fragment ions at *m/z* 185.0415. In summary, combined with standard substances and the literature, it was inferred as geniposide (Zuo et al., 2014).

#### Organic Acids and Amino Acid

Many organic acid compounds are mainly classified into small molecule phenolic acid compounds and acylated organic acid compounds according to their structure (Krzymińska et al., 2020). A total of 18 organic acid and amino acid chemical components (compound 1-6, 8-10, 12, 18-19, 21, 23, 25, 31-32, and 70) were identified in this study: quinic acid, malic acid, arginine, proline, tyrosine, citric acid, isoleucine, 3,4-dihydroxybenzaldehyde, phenylalanine, danshensu, 3-indoleacrylic acid, tryptophan, 3,5-dimethoxy-4-hydroxycinnamic acid, gentisic acid, chlorogenic acid, 4-ocaffeoylquinic acid, ferulic acid, and isochlorogenic acid. In negative ion mode, the parent ion of the small-molecule phenolic acid compound easily lost the H_2_O and CO_2_ groups, and the acylated organic acid easily produced fragment ions at *m/z* 191 [C_7_H_11_O_6_]^−^, 179 [C_9_H_7_O_4_]^−^, 173 [C_7_H_9_O_5_]^−^, 163 [C_9_H_7_O_3_]^−^, and 135 [C_8_H_7_O_2_]^−^. Similarly, for compound 25, ions at *m/z* 353.0878 [M-H]^−^, 335.0730 [M-H-H_2_O]^−^, 191.0558 [M-H-Caffeoyl]^−^, 179.0348 [M-H-Quinic]^−^, 173.0454 [M-H-Caffeoyl-H_2_O]^−^, 135.0447 [M-H-Quinic-CO_2_]^−^ were acquired in negative mode, and those at *m/z* 335.0730 [M-H-H_2_O]^−^, 191.0558 [M-H-Caffeoyl]^−^, and 179.0348 [M-H-Quinic]^−^, corresponding to NLs of 18 Da (H2O), 162 Da (caffeoyl), 174 Da (quinic), and the parent ion for *m/z* 353.0878 [M-H]^−^, were indicative of 4-ocaffeoylquinic acid (Guo et al., 2014), indicating formula C_16_H_18_O_9_. The cleavage pathway is shown in Figure S8.

#### Others

A total of 11 other components (compounds 7, 11, 16, 22, 24, 29, 36, 62, 63, 66, and 72) were identified in this study: adenosine, maltol, 7,8-dihydroxy-4-methylcoumarin, jasminoside B, eleutheroside B, norharmane, syringetin 3-glucoside, obaculactone, casticin, 3,7-dihydroxy-3′,4′-dimethoxyflavone, and β-sitosterol.

### Non-similarity Degree Evaluation of Chemical Fractions

#### Qualitative Analysis

The accumulative variance contributions of the first three principal components were 94.95% (positive ion mode) and 94.34% (negative ion mode), respectively, indicating that the PCA method achieved a higher recognition rate. As shown in Supplementary Figure S9A,B, a clear separation was observed among the four chemical fractions, indicating that the degree of similarity of the chemical fractions was small. Furthermore, HCA was performed using the data of 72 chemical components, and a clear separation was observed among the four chemical fractions as well. In addition, we intuitively understood the main components of the four chemical fractions. As shown in Supplementary Figure S10, the main components of 40AEF, 90AEF, PEF, and WEF were alkaloids, flavonoids, and a few alkaloids, iridoids, organic acids, and amino acids.

#### Quantitative Analysis

Further, the non-similarity degree (NSD) was measured by the cross-analysis of the peak areas of crude drug, angle cosine analysis method, and correlation coefficient analysis method, and was used to quantitatively analyze the non-overlapping property of the chemical fractions. As shown in Supplementary Table S2, the results showed that NSD was higher than 96.27% among the different fractions.

In this study, 72 chemical components were identified, and the main components of 40AEF, 90AEF, PEF, and WEF were alkaloids, flavonoids, alkaloids, iridoids, organic acids, and amino acids. Meanwhile, the non-overlapping property of the chemical fractions was measured by cross-analysis of the peak areas of the crude drug, angle cosine analysis method, and correlation coefficient analysis method, which was used to verify the results of the HPLC data. As shown in Supplementary Table S3, the analysis results showed that NSD was higher than 81.00% among the different fractions.

### Evaluation of Skin Lesions on AD Mice Model Treated With HLJDD and its Fractions

**FIGURE 1:**
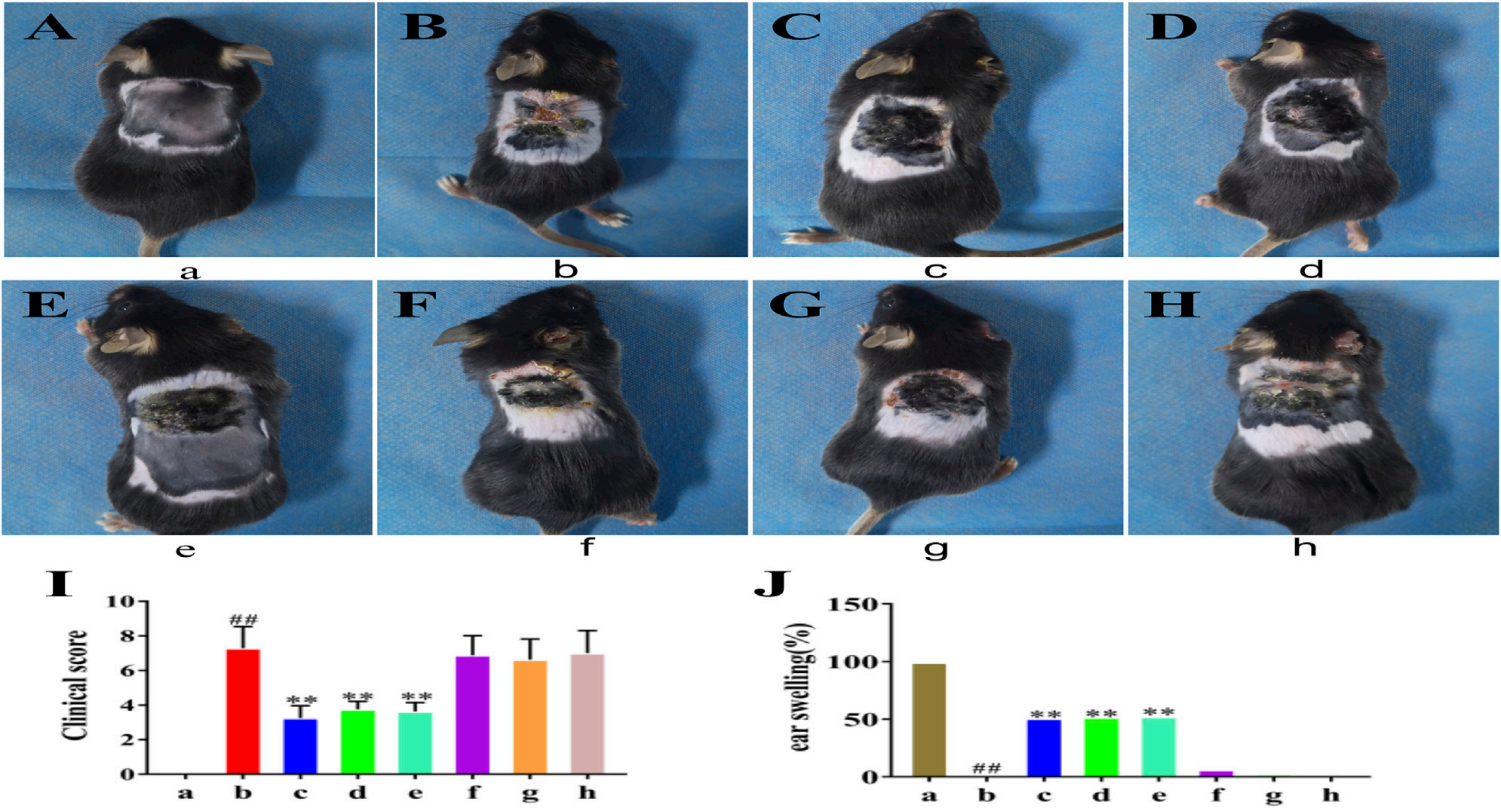
The atopic dermatitis animal model and HLJDD and its fractions treatment. (a) normal control group, (b) AD model group, (c) HLJDD group, (d) CPF group, (e) 40AEF group, (f) 90AEF group, (g) WEF group, (h) PEF group. Date was mean ± SEM (*n* = 8). ^#^ *p* < 0.05, ^##^ *p* < 0.01 *vs.* normal group; *p < 0.05, **p < 0.01 *vs*. AD model group. **(A–H)** Clinical features of dorsal skin lesions in each group; **(I)** The score of dorsal skin lesions in each group; **(J)** The difference in thickness of left and right ears of mice in each group.

The AD model was established with DNFB and used to investigate the therapeutic effects of HLJDD (12.8 g/kg) and its fractions (CPF, 40AEF, 90AEF, WEF, and PEF). In comparison with the normal control group, dorsal skin from the AD model group demonstrated severe lesions and showed typical allergic responses, including itching, increased ear and epidermal thickness, swelling, dryness, and erythema. After treatment, the above symptoms in the dorsal skin of mice in the HLJDD group and groups treated with its fractions were remarkably improved (Figures 1A–H), especially in the HLJDD, 40AEF, and 90AEF groups. Furthermore, the results of clinical scores indicated that the scores of the AD model group were significantly higher than those of the normal control group, while the HLJDD, CPF, and 40AEF groups showed significantly lower scores (*p* < 0.01) (Figure 1I). In addition, the inhibitory rate of ear swelling was significantly increased in the HLJDD, CPF, and 40AEF groups compared to the AD model group (Figure 1J). These results indicated that HLJDD and its fractions had significant ameliorating effects on AD model mice, especially HLJDD, 40AEF, and CPF.

### Histological Examination

**FIGURE 2:**
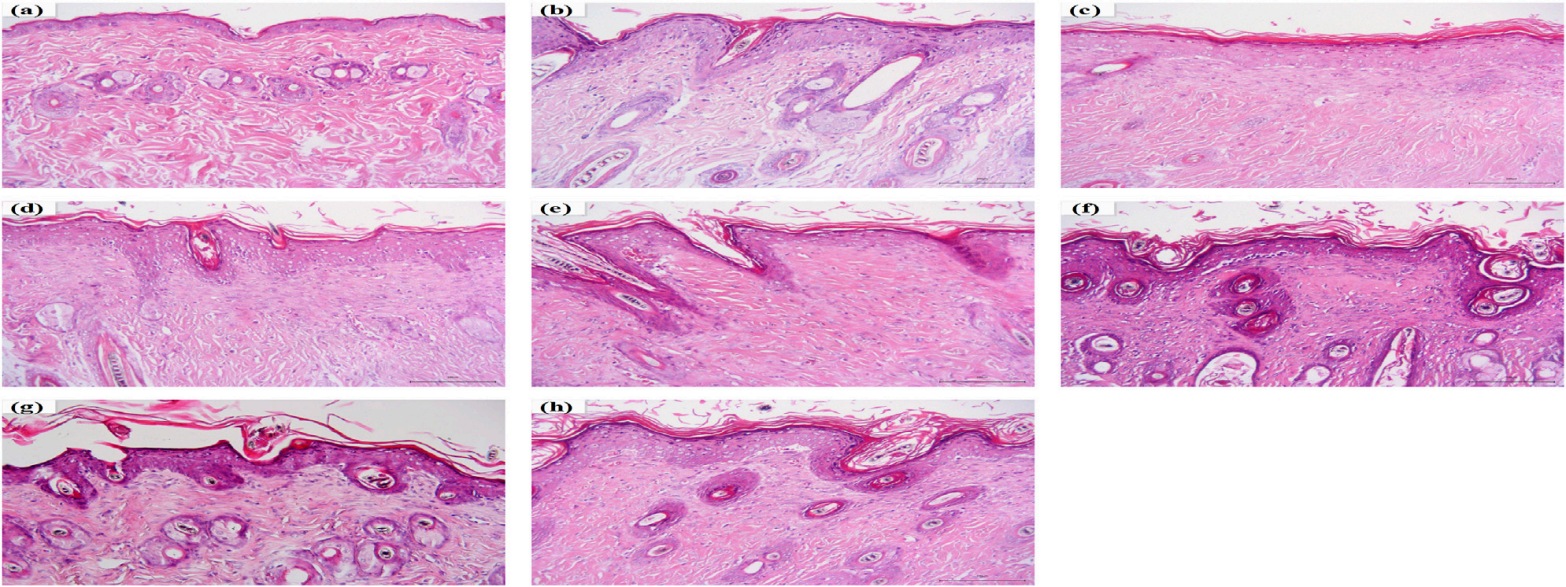
Hematoxylin and eosin (H&E) staining. **(A)** normal control group, **(B)** AD model group, **(C)** HLJDD group, **(D)** CPF group, **(E)** 40AEF group, **(F)** 90AEF group, **(G)** WEF group, **(H)** PEF group.

To further demonstrate the therapeutic effect of HLJDD and its fractions, we compared the pathology of DNFB-induced skin lesions with or without HLJDD and its fractions (Figure 2). In the normal control group, the subcutaneous structure was intact and lined with a thin epithelial layer, showing no signs of inflammatory cell infiltration. In the AD model group, thickening of the epidermis was obvious, where hyperkeratosis and thickening of the spinous cell layer had occurred. Inflammatory cells infiltrating the dermis were also observed in the AD model group. A similar pathology was observed in the 90AEF, WEF, and PEF groups, however, the thickening of epidermis and the infiltration of inflammatory cells were significantly reduced in the HLJDD, CPF, and 40AEF groups, suggesting that HLJDD, CPF, and 40AEF could indeed improve DNFB-induced skin lesions.

### Analysis of HLJDD and its Fractions on Total Serum IgE, Histamine, and Relative Cytokines

**FIGURE 3:**
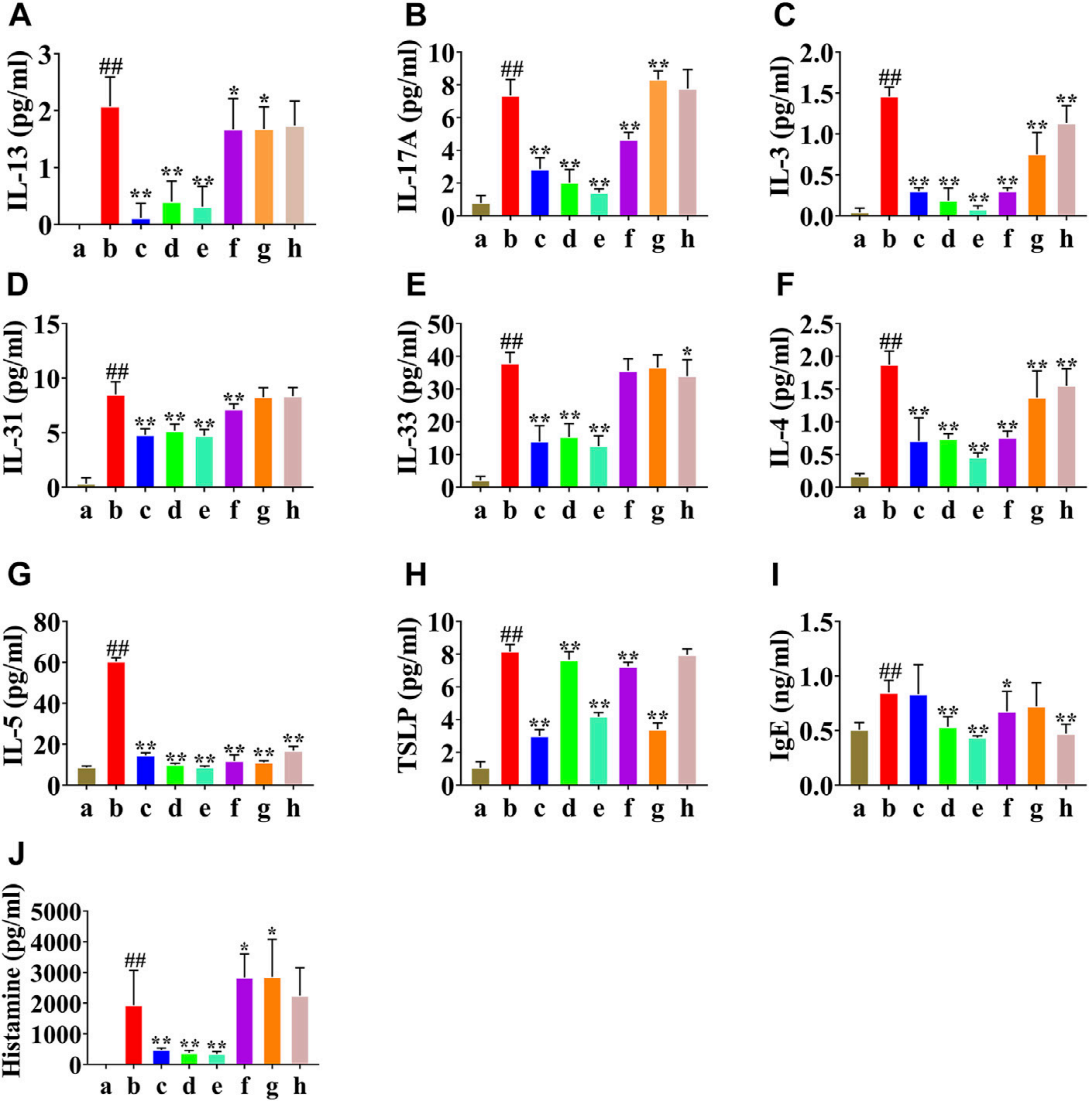
The atopic dermatitis animal model and HLJDD and its fractions treatment **(A–J)**. (a) normal group, (b) AD model group, (c) WD group, (d) CPF (300 mg/kg) group, (e) 40AEF group, (f) 90AEF group, (g) WEF group, (h) PEF group. Date was mean ± SEM (*n* = 8). ##*p* < 0.01 vs. normal group; *p < 0.05, **p < 0.01 vs. AD model group.

To assess the therapeutic effects of HLJDD and its fractions on AD model mice, the total serum IgE and histamine levels were detected by ELISA, and the levels of IL-13, IL-17A, IL-3, IL-31, IL-33, IL4, IL-5, and TSLP were detected using Luminex 200™. The serum IL-13, IL-17A, IL-3, IL-31, IL-33, IL4, IL-5, TSLP, IgE, and histamine levels were significantly increased in the AD model group compared to the normal control group. However, HLJDD markedly decreased serum IL-13, IL-17A, IL-3, IL-31, IL-33, IL4, IL-5, TSLP, and histamine levels; CPF and 40AEF markedly decreased serum IL-13, IL-17A, IL-3, IL-31, IL-33, IL4, IL-5, TSLP, IgE, and histamine levels; 90AEF markedly decreased serum IL-13, IL-17A, IL-3, IL-31, IL4, IL-5, TSLP, IgE, and histamine levels; WEF markedly decreased serum IL-13, IL-17A, IL-3, IL4, IL-5, TSLP, and histamine levels; PEF markedly decreased serum IL-3, IL-33, IL4, IL-5, and IgE levels. These results indicate that HLJDD and its fractions (especially HLJDD, 40AEF, and 90AEF) had significant ameliorating effects on AD model mice by downregulating the total serum IgE, histamine, and relative cytokines (Figures 3A–J).

### The Effect of HLJDD and its Fractions on HRH4, IL-4Rα, and JAK1 mRNA Levels

**FIGURE 4:**
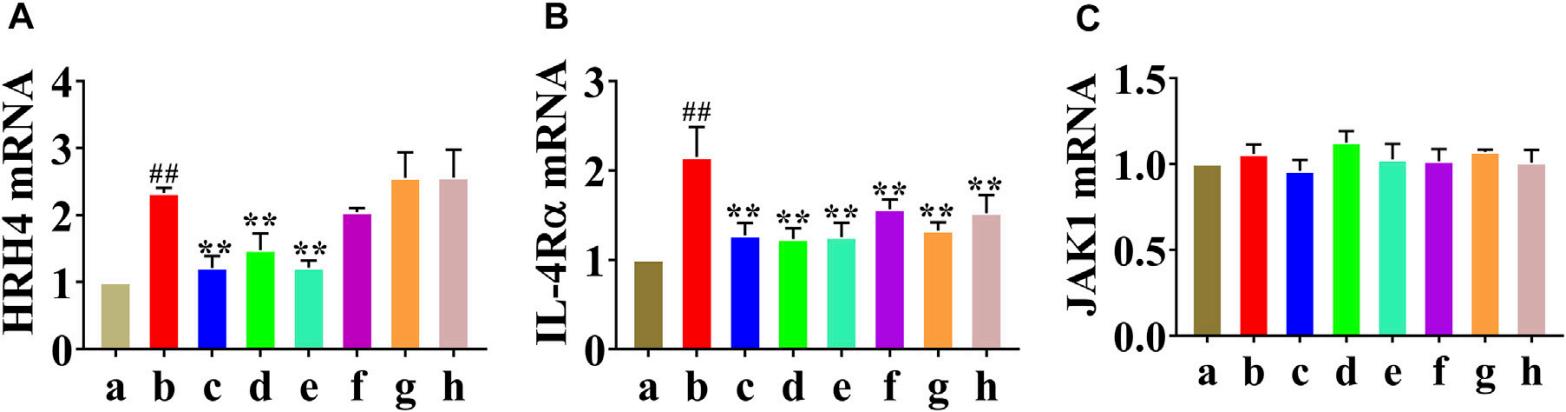
Effects of HLJDD and its fractins on mRNA expression of HRH4 **(A)**, IL-4Rα **(B)**, and JAK1 **(C)** in the mice. (a) normal control group, (b) AD model group, (c) HLJDD group, (d) CPF group, (e) 40AEF group, (f) 90AEF group, (g) WEF group, (h) PEF group. Date was mean ± SEM (*n* = 8). ^#^ *p* < 0.05, ^##^ *p* < 0.01 *vs.* normal group; *p < 0.05, **p < 0.01 *vs*. AD model group.

Real-time PCR analysis was performed to examine whether HLJDD affected HRH4, IL-4Rα, and JAK1 mRNA expression. As shown in Figure 4A, the relative content of HRH4 mRNA in the AD model group was increased compared to that in the normal control group (*p* < 0.01), and the HLJDD, CPF, and 40AEF groups were significantly decreased compared with the AD model group (*p* < 0.01). As illustrated in Figure 4B, the relative content of IL-4Rα mRNA in the AD model group was increased compared to that in the normal control group (*p* < 0.01), and the HLJDD, CPF, 40AEF, 90AEF, WEF, and PEF groups were significantly decreased compared with the AD model group (*p* < 0.01). However, as illustrated in Figure 4C, HLJDD and its fractions did not show an inhibitory effect on the expression of JAK1 mRNA.

### HLJDD and its Fractions Decreased HRH4, IL-4Rα, and JAK1 Protein Expression in AD Model Mice

**FIGURE 5:**
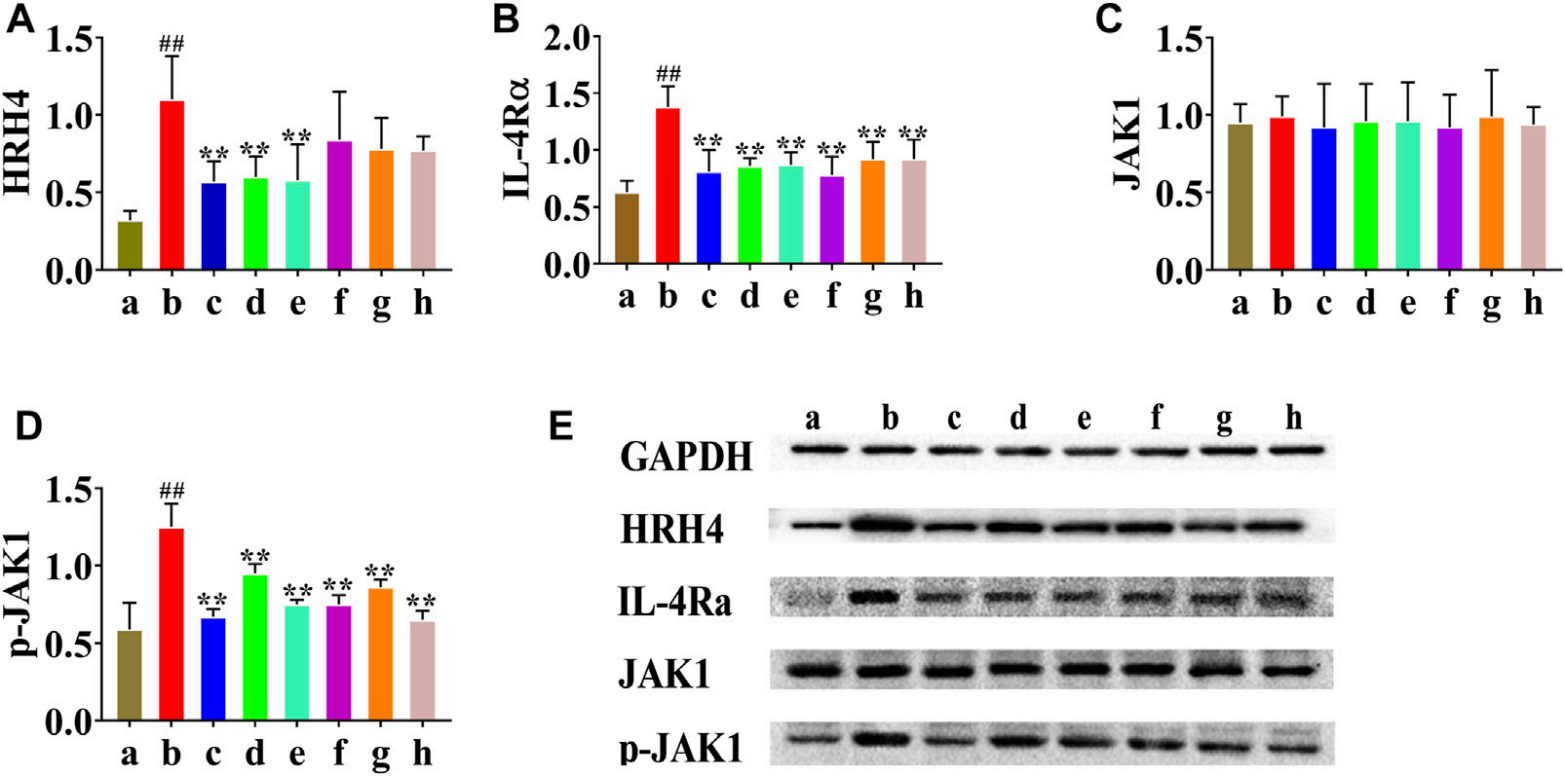
Effects of HLJDD and its fractions on protein expression of HRH4, IL-4Rα, JAK1, and p-JAK1 in dorsal skin tissues. The protein expression of HRH4, IL-4Rα, JAK1, and p-JAK1 were detected by Western blotting, and quantified by densitometry **(E)**. Relative expressions of HRH4, IL-4Rα, JAK1 and p-JAK1 were normalized to GAPDH internal control **(A–D)**. Data were presented as the means ± SEM, ##*p* < 0.01 vs. normal group; *p < 0.05, **p < 0.01 vs. AD model group. (a) normal group, (b) AD model group, (c) WD group, (d) CPF group, (e) 40AEF group, (f) 90AEF group, (g) WEF group, (h) PEF group.

In addition, HRH4, IL-4Rα, JAK1, and p-JAK1 protein expression was detected by western blotting. As shown in Figures 5A–E, HRH4, IL-4Rα, and p-JAK1 protein expression was significantly upregulated in the AD model group (*p* < 0.01). After treatment, HLJDD, CPF, and 40AEF significantly reduced the levels of HRH4, IL-4Rα, and p-JAK1 (*p* < 0.01), and 90AEF, WEF, and PEF significantly reduced the levels of IL-4Rα and p-JAK1 (*p* < 0.01). Interestingly, there were no significant changes in the protein expression of JAK1.

### Untargeted Metabolomics

#### PLS-DA Analysis

**FIGURE 6:**
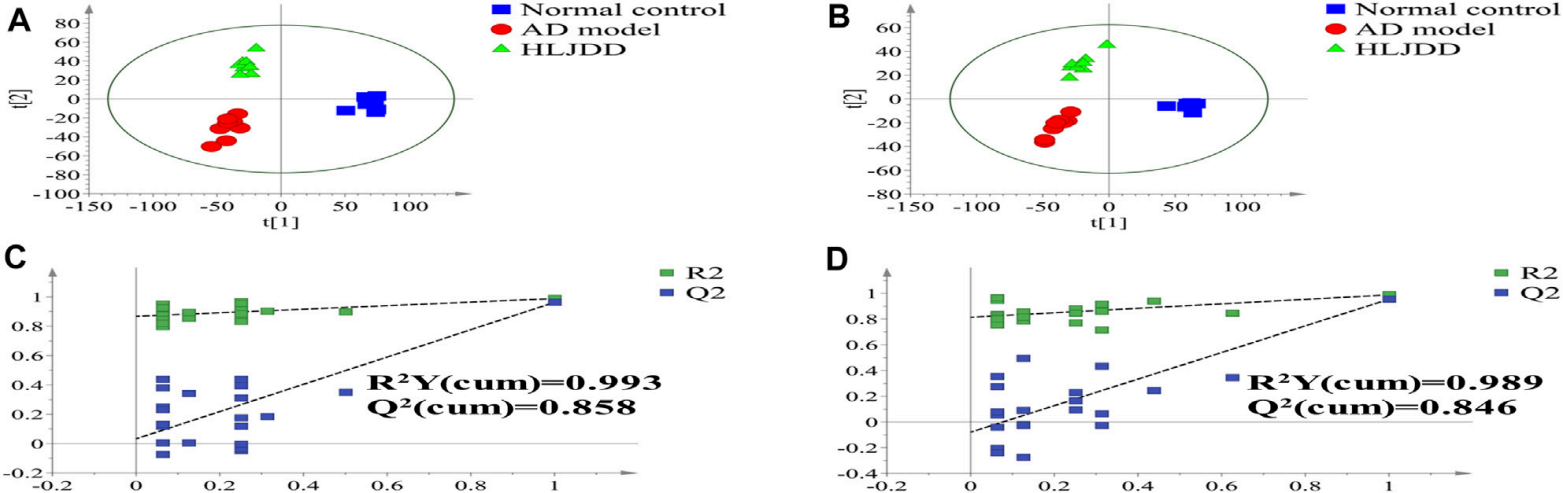
PLS-DA score chart of mice serum samples in positive and negative modes. **(A)** PLS-DA score plots in positive mode, **(B)** PLS-DA score plots in negative mode, **(C)** permutation plots of the PLS-DA models in positive mode, **(D)** permutation plots of the PLS-DA models in negative mode.

To identify chemical differences between high-dimensional mass spectra data measured for different groups, we used partial least squares discrimination analysis (PLS-DA) to evaluate the metabolomics datasets. In the PLS-DA analysis, for the positive ion mode, R^2^Y (cum) = 0.993 and Q^2^ (cum) = 0.858; for the negative ion mode, R^2^Y (cum) = 0.989 and Q^2^ (cum) = 0.846. Both *R* ^2^ and Q^2^ exceeded 0.5, indicating that the statistical model had a good fit and predictive ability. There were differences between the three major clusters: the AD model group was set aside from the normal group and the HLJDD group (Figures 6A–D). Therefore, the metabolite profiles were distinctively altered by treatment with HLJDD, suggesting that HLJDD can be an effective treatment for AD. This result is also consistent with the experimental data, where HLJDD showed significant therapeutic efficacy in AD models. Additionally, we observed that the metabolite profile of the control group was very different from that of the model group, suggesting that AD may have caused irreversible metabolic disorders in animals.

#### Identification of Potential Biomarkers

Biomarkers were screened according to a threshold of variable importance in the projection (VIP) value (VIP > 1) that was generated after PLS-DA processing. The *p*-value for significantly differential metabolites was set to 0.05. According to the above criteria, 40 significant biomarkers were identified and selected for further study (Table 2). Compared with normal control group, the levels of 25 metabolites (L-alloisoleucine, 2-hydroxy-2-methylbutanedioic acid, linolelaidic acid, (2S)-2-aminopentanedioic acid, L-alanine, carnosine, (Z)-tetracos-15-enoic acid, malic acid, gamma-linolenic acid, octadecanedioic acid, (5Z,8Z,11Z,14Z,16E)-18-hydroxyicosa-5,8,11,14,16-pentaenoic acid, D-phenyllactic acid, deoxyuridine, 2-(5-hydroxy-1H-indol-3-yl)acetic acid, orotic acid, prostaglandin D1, N′-hydroxymethylnorcotinine, biliverdin, lysoPE (20:4 (8Z,11Z,14Z,17Z)/0:0), N-[(3R,4R,5S,6R)-2,4,5-trihydroxy-6-(hydroxymethyl)oxan-3-yl]acetamide, N-a-acetyl-L-arginine, L-hypoglycin A, dihydro-O-methylsterigmatocystin, 6-lactoyltetrahydropterin, and phosphocreatinine) were significantly upregulated and 15 metabolites (N-methyl-D-aspartic acid, 3,7-dimethylpurine-2,6-dione, indole-3-propionic acid, 1H-pyrimidine-2,4-dione, docosahexaenoic acid, L-glutamic acid, gamma-glutamylvaline, lysoPC(24:1 (15Z)), humulinone, 4-imidazolone-5-propionic acid, alanyl-glutamine, PC(P-18:1 (11Z)/20:5 (5Z,8Z,11Z,14Z,17Z)), cytosine, glutaminylphenylalanine, and gamma-glutamylleucine)) were significantly downregulated in the AD model group. After treatment, these metabolites were restored to varying degrees in the HLJDD group.

**TABLE 2:** Mass databases of 40 potential differential metabolites.

No	R.T.(s)	*m/z*	Ion mode	Identification results (MS2)	Control group (peak area abundance)	Model group (peak area abundance)	HJDD (peak area abundance)	VIP
1	311.56	131.0356	N	L-Alloisoleucine	14.21 ± 2.31	6.43 ± 1.7^##^	8.39 ± 1.29^*^	1.19
2	353.64	146.0824	N	2-hydroxy-2-methylbutanedioic acid	3.1 ± 0.92	5.67 ± 0.67^##^	4.83 ± 0.56^*^	1.19
3	45.51	279.2325	N	Linolelaidic acid	384.69 ± 50.49	674.06 ± 65.02^##^	512.09 ± 133.43**	1.19
4	404.55	146.0460	N	(2S)-2-aminopentanedioic acid	42.57 ± 4.46	50.39 ± 9.34^##^	41.51 ± 6.68**	1.24
5	349.66	88.0420	N	L-Alanine	21.34 ± 3.27	28.47 ± 5.13^##^	22.66 ± 5.56^*^	1.30
6	384.33	226.0482	N	Carnosine	0.95 ± 0.16	1.82 ± 0.1^##^	1.14 ± 0.32**	1.32
7	313.56	146.0461	N	N-Methyl-D-aspartic acid	22.14 ± 3.37	13.39 ± 1.49^##^	18.32 ± 4.08**	1.04
8	272.39	179.0558	N	3,7-dimethylpurine-2,6-dione	39.29 ± 9.98	15.91 ± 4.57^##^	22.15 ± 6.17^*^	1.14
9	26.91	365.0324	N	(Z)-tetracos-15-enoic acid	7.56 ± 4.22	13.47 ± 2.94^##^	6.51 ± 1.58**	1.15
10	423.32	132.0308	N	Malic acid	1.62 ± 0.68	6.15 ± 2.29^##^	2.98 ± 1.04**	1.27
11	95.55	187.1338	N	Indole-3-propionic acid	6.53 ± 2.11	3.67 ± 2.9^##^	7.32 ± 3.14**	1.06
12	324.89	110.0991	N	1H-pyrimidine-2,4-dione	0.44 ± 0.07	0.16 ± 0.08^##^	0.32 ± 0.15**	1.34
13	45.69	278.2204	N	γ-Linolenic acid	11.87 ± 2.45	23.29 ± 3.3^##^	17.32 ± 6.13**	1.13
14	98.97	327.1076	N	Docosahexaenoic acid	6.52 ± 2.9	3.91 ± 1.13^#^	10.94 ± 4.37**	1.38
15	384.81	131.0547	N	L-Glutamic acid	3.84 ± 0.63	1.53 ± 0.6^##^	2.32 ± 0.3**	1.23
16	77.65	313.2376	N	octadecanedioic acid	5.46 ± 1.24	9.35 ± 4.51^##^	4.71 ± 1.19**	1.17
17	39.25	317.1903	N	(5Z,8Z,11Z,14Z,16E)-18-hydroxyicosa-5,8,11,14,16-pentaenoic acid	0.24 ± 0.14	1.31 ± 0.53^##^	0.76 ± 0.27**	1.28
18	109.13	165.0145	N	D-Phenyllactic acid	3.62 ± 1.47	9.1 ± 3.46^##^	5.23 ± 3.09**	1.17
19	151.45	226.1446	N	Deoxyuridine	1.84 ± 0.36	5.41 ± 0.99^##^	3.61 ± 1.61**	1.22
20	186.79	189.0665	N	2-(5-hydroxy-1H-indol-3-yl)acetic acid	0.38 ± 0.11	2.69 ± 0.97^##^	1.8 ± 0.72**	1.13
21	244.55	155.0102	N	Orotic acid	2.55 ± 0.71	7.55 ± 3.37^##^	5.49 ± 1.99*	1.39
22	115.78	353.2324	N	Prostaglandin D1	3.53 ± 1.82	19.11 ± 3.56^##^	15.88 ± 1.96*	1.26
23	405.92	246.1332	P	gamma-Glutamylvaline	3.93 ± 0.99	2.01 ± 0.54^##^	2.89 ± 0.46**	1.23
24	189.75	606.4496	P	LysoPC(24:1 (15Z))	9.97 ± 0.72	7.93 ± 1.49^##^	9.34 ± 1.06^*^	1.32
25	294.90	192.9058	P	N′-Hydroxymethylnorcotinine	2.35 ± 0.25	3.28 ± 0.65^##^	2.64 ± 0.2^*^	1.05
26	204.67	582.3182	P	Biliverdin	1.64 ± 0.33	2.72 ± 0.36^##^	1.68 ± 0.37**	1.24
27	403.25	287.1212	P	Humulinone	4.59 ± 0.63	2.58 ± 0.63^##^	4.15 ± 0.96**	1.37
28	373.29	157.0599	P	4-Imidazolone-5-propionic acid	17.26 ± 2.67	9.13 ± 2.32^##^	15 ± 2.76**	1.36
29	203.46	502.2923	P	LysoPE (20:4 (8Z,11Z,14Z,17Z)/0:0)	42.07 ± 12.12	174.45 ± 19.51^##^	148.54 ± 21**	1.34
30	282.69	221.1206	P	N-[(3R,4R,5S,6R)-2,4,5-trihydroxy-6-(hydroxymethyl)oxan-3-yl]acetamide	0.8 ± 0.13	1.2 ± 0.31^##^	0.97 ± 0.21^*^	1.05
31	321.38	218.0418	P	Alanyl-Glutamine	1.66 ± 0.28	1.22 ± 0.17^##^	1.74 ± 0.28**	1.35
32	93.14	790.5752	P	PC(P-18:1 (11Z)/20:5 (5Z,8Z,11Z,14Z,17Z))	5.53 ± 1.7	3.54 ± 0.78^##^	6.48 ± 1.92**	1.29
33	216.41	112.0505	P	Cytosine	7.16 ± 0.74	4.83 ± 1.47^##^	7.02 ± 0.34**	1.49
34	394.61	217.0658	P	N-a-Acetyl-L-arginine	0.11 ± 0.06	0.19 ± 0.02^##^	0.12 ± 0.03**	1.29
35	434.06	294.1542	P	Glutaminylphenylalanine	6.9 ± 1.3	4.89 ± 1.58^##^	6.57 ± 1.57*	1.08
36	417.04	261.1436	P	gamma-Glutamylleucine	9.44 ± 2.92	6.57 ± 1.56^##^	8.71 ± 2.98*	1.15
37	407.92	142.0859	P	L-Hypoglycin A	1.99 ± 0.26	4.81 ± 1.74^##^	3.48 ± 0.49*	1.02
38	309.92	341.8941	P	Dihydro-O-methylsterigmatocystin	0.56 ± 0.21	1.01 ± 0.25^##^	0.72 ± 0.2**	1.47
39	496.99	240.1098	P	6-Lactoyltetrahydropterin	0.65 ± 0.1	1.49 ± 0.53^##^	1.07 ± 0.53*	1.11
40	213.38	194.0358	P	Phosphocreatinine	0.35 ± 0.15	0.94 ± 0.19^##^	0.62 ± 0.23*	1.17

Compared to control group, ^#^ *p* < 0.05, ^##^ *p* < 0.01; Compared to model group, *p < 0.05, **p < 0.01; N: negative mode, P: positive mode.

**FIGURE 7:**
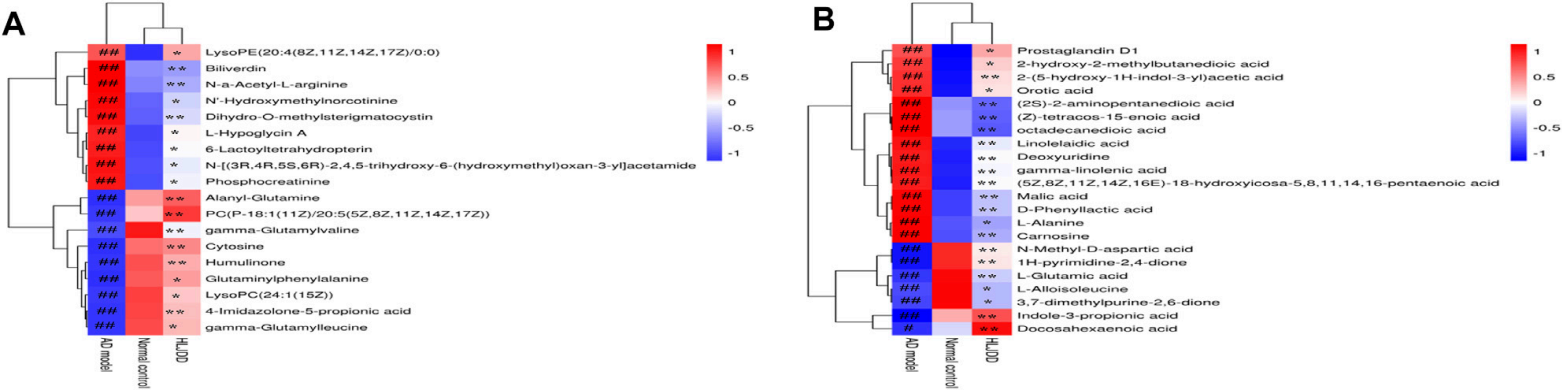
Hierarchical clustering results of 40 metabolites in ESI+ **(A)** and ESI− **(B)** mode. Note: The standard concentration was the abscissa, with the metabolites name regarded as the ordinate. Each of lattice represents a concentration in corresponding sample. The color ranges from blue to red and the shades of color represent different concentration magnitudes. ^#^ *p* < 0.05, ^##^ *p* < 0.01 *vs.* normal group; *p < 0.05, **p < 0.01 *vs*. AD model group.

Additionally, clustering heatmaps were used to show an intuitive visualization of the difference and clustering degree of metabolites among the three groups. Heatmaps of 40 metabolites were constructed and visualized using MetaboAnalyst software. As shown in Figure 7, the metabolites in the AD model group, normal control group, and HLJDD group achieved distinct clusters.

#### Metabolic Pathway Analysis

**FIGURE 8:**
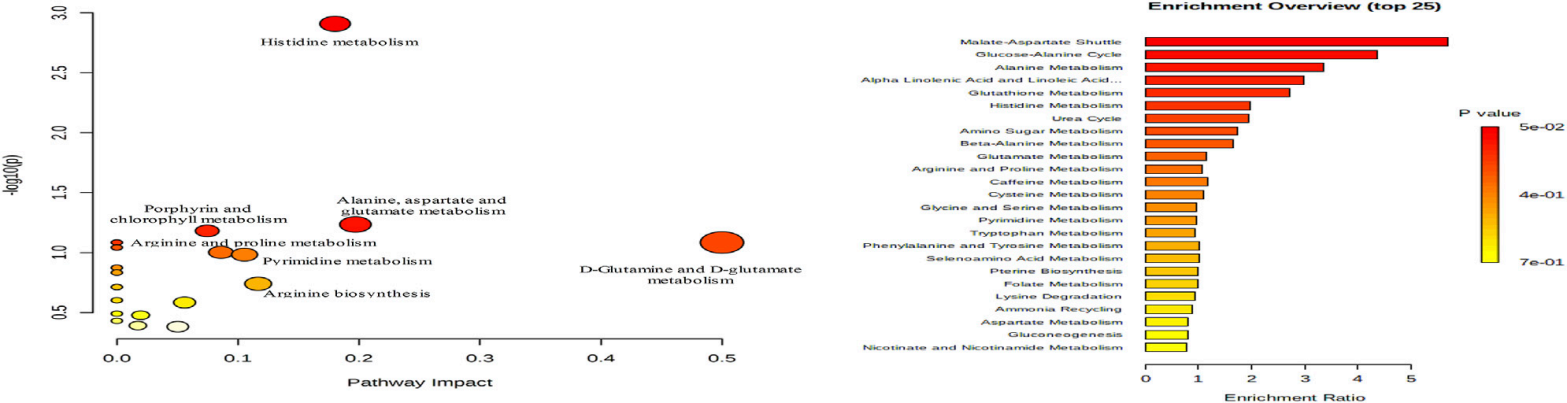
Pathway analysis of 40 potential biomarkers with MetPA. 1. Nitrogen metabolism, 2. Biosynthesis of unsaturated fatty acids, 3. Caffeine metabolism, 4. Aminoacyl-tRNA biosynthesis, 5. Butanoate metabolism, 6. Pantothenate and CoA biosynthesis, 7. Selenocompound metabolism, 8. Folate biosynthesis, 9. Glyoxylate and dicarboxylate metabolism, 10. Glutathione metabolism, 11. Glycerophospholipid metabolism, 12. Tryptophan metabolism, 13. Amino sugar and nucleotide sugar metabolism, 14. beta-Alanine metabolism, 15. Porphyrin and chlorophyll metabolism, 16. Arginine and proline metabolism, 17. Arginine biosynthesis, 18. Histidine metabolism, 19. Pyrimidine metabolism, 20. Alanine, aspartate and glutamate metabolism, 21. D-Glutamine and D-glutamate metabolism.

Metabolic pathway analysis was performed with MetPA using the identified biomarkers as input, which revealed 21 metabolic pathways that may be involved in the dysregulation of metabolism in AD (Figure 8). Pathways with impact values over 0.1 were considered as candidate target pathways. Among them, four pathways, namely, histidine metabolism, arginine biosynthesis, pyrimidine metabolism, and alanine, aspartic acid, and glutamate metabolism, have been suggested to be subject to perturbation by HLJDD to improve the endogenous metabolism of AD.

### Targeted Metabolomics

#### Selection of Metabolites for Targeted Metabolomics and Construction of a Standard Curves

**FIGURE 9:**
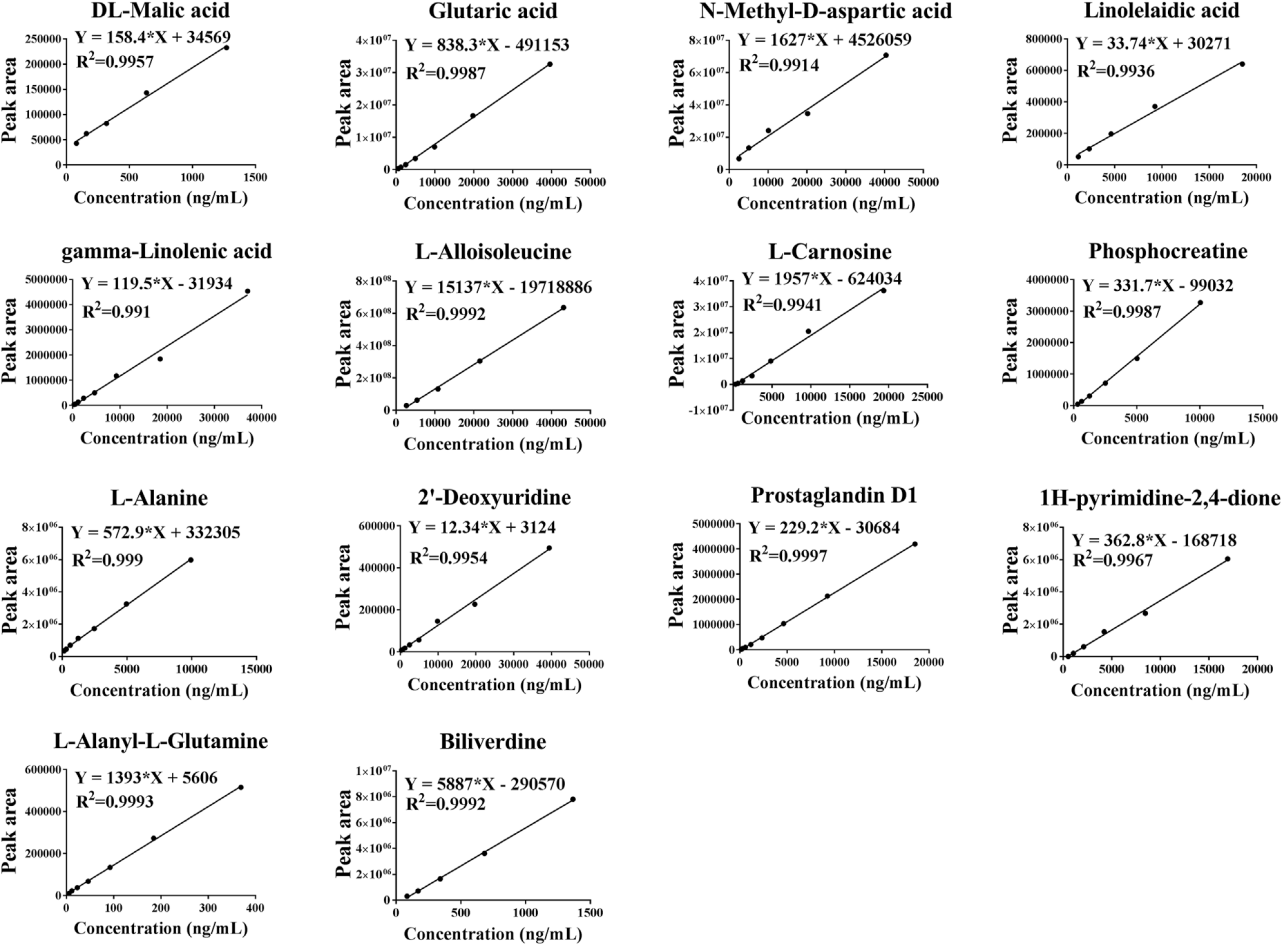
Standard curves of 14 different metabolites of DL-Malic acid, Glutaric acid, N-Methyl-D-aspartic acid, linolelaidic acid, γ-Linolenic acid, L-Alloisoleucine, L-Carnosine, phosphocreatinine, L-Alanine, 2′-Deoxyuridine, Prostaglandin D1, 1H-pyrimidine-2,4-dione, L-Alanyl-L-Glutamine, and Biliverdine.

For the screening diagnostic biomarkers, pearson’s correlation analysis was used for the correlation analysis of 17 physiological and biochemical indices (skin lesion score, IL-13, IL-17, IL-3, IL-31, IL-33, IL-4, IL-5, TSLP, histamine, HRH4 mRNA, IL-4Rα mRNA, JAK1 mRNA, IgE, IL-4Rα, JAK1, p-JAK1, and HRH4) and 40 potential biomarkers. First, in the normal control group and AD model group, a total of 190 metabolites were significantly associated with physiological and biochemical indices (Supplementary Figure S11), which could act as diagnostic biomarkers for AD. Second, in the AD model group and HLJDD group, a total of 190 metabolites were significantly associated with macroscopic indexes (Supplementary Figure S12), which could act as a potential biomarker for AD treated with HLJDD. To increase the diagnostic capability of the potential biomarkers, we took the intersection of the two sets of predictions. Finally, 23 metabolites (prostaglandin D1, phosphocreatinine, n-methyl-d-aspartic acid, n-hydroxymethylnorcotinine, malic acid, lysopc (24:1 (15z)), linolelaidic acid, l-alloisoleucine, l-alanine, humulinone, glutaric acid, gamma-linolenic acid, dihydro-o-methylsterigmatocystin, deoxyuridine, d-phenyllactic acid, carnosine, biliverdin, alanyl-glutamine, 4-imidazolone-5-propionic acid, 3,7-dimethylpurine-2,6-dione, 2-hydroxy-2-methylbutanedioic acid, 1h-pyrimidine-2,4-dione, and (z)-tetracos-15-enoic acid) were selected as diagnostic biomarkers of AD. Considering the accuracy of metabolites and the diversity detected in each sample, 14 endogenous metabolites (DL-malic acid, glutaric acid, N-methyl-D-aspartic acid, linolelaidic acid, gamma-linolenic acid, L-alloisoleucine, L-carnosine, phosphocreatinine, L-alanine, 2′-deoxyuridine, prostaglandin D1, 1H-pyrimidine-2,4-dione, L-alanyl-L-glutamine, and biliverdine) were selected for targeted metabolomics analysis. As shown in Figure 9, a standard curve of 14 endogenous metabolites was established. The curve fit all values of *R* ^*2*^ > 0.9, indicating that the concentration calculation based on these curves was accurate.

#### Assessment of 14 Metabolites in HLJDD and its Fractions by Targeted Metabolomics

**FIGURE 10:**
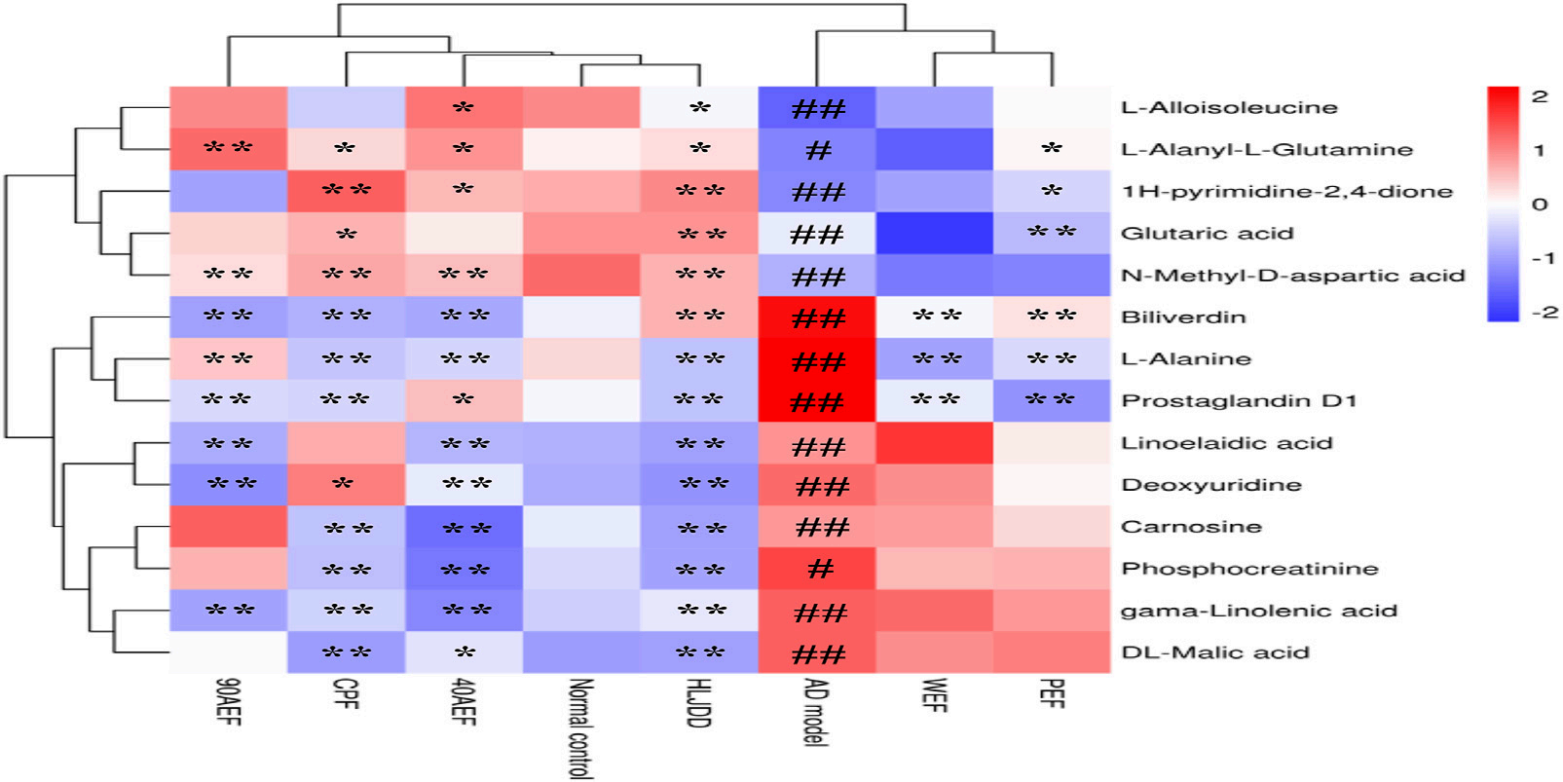
Hierarchical clustering results of 14 metabolites in target metabolomics. Note: The standard concentration was the abscissa, with the metabolites name regarded as the ordinate. Each of lattice represents a concentration in corresponding sample. The color ranges from blue to red and the shades of color represent different concentration magnitudes. ^#^ *p* < 0.05, ^##^ *p* < 0.01 *vs.* normal group; *p < 0.05, **p < 0.01 *vs*. AD model group.

As shown in Figure 10, compared with the normal control group, L-alloisoleucine, L-alanyl-L-glutamine, glutaric acid, 1H-pyrimidine-2,4-dione, and N-methyl-D-aspartic acid were significantly downregulated, and glutaric acid, N-methyl-D-aspartic acid, linolelaidic acid, gamma-linolenic acid, L-carnosine, phosphocreatinine, L-alanine, 2′-deoxyuridine, prostaglandin D1, L-alanyl-L-glutamine, biliverdine, and DL-malic acid were significantly upregulated in the AD model group. After treatment, these metabolites were restored to varying degrees in the HLJDD group, which was also consistent with the findings of the untargeted metabolomics analysis. Meanwhile, we found that these metabolites were also restored to varying degrees in the CPF, 40AEF, 90AEF, WEF, and PEF groups, indicating that CPF, 40AEF, 90AEF, WEF, and PEF exhibited different degrees of therapeutic activity against AD, especially HLJDD, 40AEF, and CPF. Hierarchical clustering analysis was performed using MetaboAnalyst software, the results of which revealed that there were two clusters (AD model group, WEF, PEF; normal control group, HLJDD, CPF, 40AEF, 90AEF), which further validated the therapeutic effects of HLJDD and its fractions, especially HLJDD, 40AEF, and CPF. Therefore, 40AEF and CPF are considered the most important components of HLJDD for ameliorating AD.

#### Evaluation of Diagnostic Efficacy of Potential Biomarkers

**FIGURE 11:**
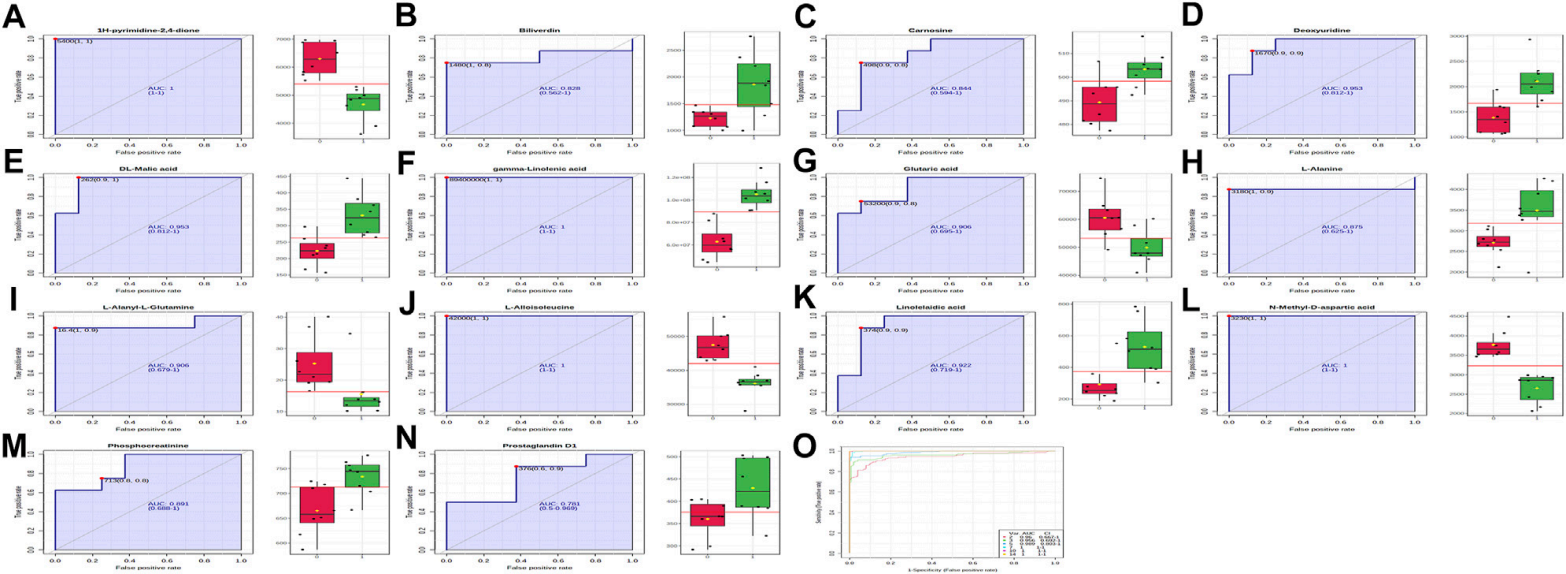
Classical univariate and multivariate ROC curve analyses. **(A)**, 1H-pyrimidine-2,4-dione (AUC = 1); **(B)**, biliverdine (AUC = 0.828); **(C)**, carnosine (AUC = 0.844); **(D)**, deoxyuridine (AUC = 0.953); **(E)**, DL-malic acid (AUC = 0.953); **(F)**, gamma-linolenic acid (AUC = 1); **(G)**, glutaric acid (AUC = 0.906); **(H)**, L-alanine (AUC = 0.875); **(I)**, L-alanyl-L-glutamine (AUC = 0.906); **(J)**, L-alloisoleucine (AUC = 1); **(K)**, linolelaidic acid (AUC, 0.922); **(L)**, N-methyl-D-aspartic acid (AUC, 1); **(M)**, Phosphocreatine (AUC = 0.891); **(N)**, prostaglandin D1 (AUC = 0.781); **(O)**, multivariate ROC curve analyses.

ROC curves were used to assess the sensitivity and specificity of the diagnostic performance of potential biomarkers (Hao et al., 2010; Li, et al., 2014a). Diagnostic accuracy was analyzed by area under curve (AUC) of ROC, and AUC was considered to be of good accuracy when AUC > 0.7 (Abdel-Gawad et al., 2009). Classical univariate ROC curve analyses and multivariate ROC curve based exploratory analysis (Explorer) were performed using MetaboAnalyst 5.0 (https://www.metaboanalyst.ca/). The results of classical univariate ROC curve analyses indicated that the AUC of 14 metabolites was greater than 0.7 (Figures 11A–N). The results of the multivariate ROC curve based exploratory analysis indicated that the AUC was greater than 0.9 (Figure 11O). Therefore, these metabolites may be potential candidate biomarkers for predicting AD.

## Discussion

HLJDD is a traditional Chinese medicine composed of four herbs, *Coptis chinensis* Franch., *Scutellaria baicalensis* Georgi, *Phellodendron amurense* Rupr., and *Gardenia jasminoides* J. Ellis. It is frequently prescribed due to its effectiveness against serious illness, including those of the skin (Chen et al., 2017; Chen et al., 2016). With these properties, HLJDD has a curative effect in the treatment of AD (Qi et al., 2019). Clinical and experimental studies have confirmed that HLJDD is an effective drug for the treatment of AD. However, the effective ingredients and underlying molecular mechanisms have not yet been fully explored (Kobayashi et al., 2004). Previous studies have shown that the major constituents of HLJDD include alkaloids, flavonoids, iridoids, organic acids, and amino acids (Wang et al., 2020). Therefore, further research is needed to elucidate the possible active ingredients responsible for the anti-AD action of HLJDD.

Traditional Chinese medicine always exhibits “multi-component” and “multi-target” characteristics in the treatment of diseases, which is a big challenge to clarify the pharmacodynamic components of Chinese drugs. In general, decoctions or alcoholic extracts of Chinese drugs were split into multiple fractions, which were used to clarify its substance basis (Li et al., 2014a). Based on previous laboratory research (Chen et al., 2021; Han et al., 2020), we developed a reliable method for the rapid screening of chemical constituents and the evaluation of non-similarity degrees of splitting fractions of HLJDD using HPLC and UPLC-Q-TOF/MS/MS techniques. In this study, HLJDD was split into five fractions, and a total of 72 chemical constituents were analyzed and identified, where the different fractions showed significant chemical differences (>81%). The main components of 40AEF, 90AEF, PEF, and WEF were alkaloids, flavonoids, and a few alkaloids, iridoids, organic acids, and amino acids, respectively, which provides insights into understanding the complex compounds of HLJDD and further analyzing the pharmacological studies of different fractions.

**FIGURE 12:**
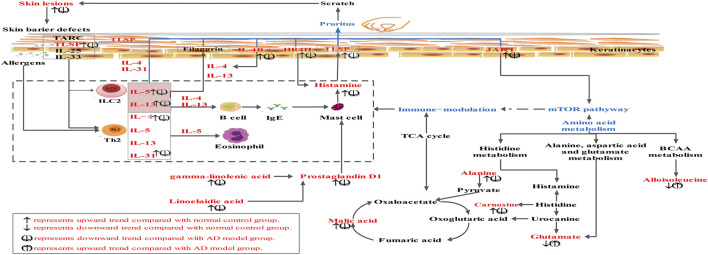
The amelioration mechanism of HLJDD and its fractions in AD. Compared with normal control group, “↑” represents upward trend in normal group, “↓” represents downward trend in normal group. Compared with AD model group, “(↑)” represents upward trend in HLJDD group and its fractions groups, “(↓)” represents downward trend in HLJDD group and its fractions groups.

Furthermore, a DNFB-induced AD mouse model was used to explore the therapeutic effects of HLJDD and its fractions on AD. Owing to its simplicity and ease of use, high repeatability, and easy quantization of clinical scores, the model has been widely used for anti-AD drug discovery and development (Shiohara et al., 2004; Xiong et al., 2020; Zeng et al., 2021). Our results showed that typical allergic responses, including itching, increased ear and epidermal thickness, swelling, dryness, and erythema were observed in the AD model, which was consistent with a previous study (Xiong et al., 2020). However, the above symptoms in the dorsal skin of mice in the HLJDD group and the groups treated with its fractions were remarkably improved. The results indicated that HLJDD and its fractions had therapeutic effects on AD, especially 40AEF and CPF. Furthermore, combined with the results of physiological and biochemical indices and metabolomics, the amelioration mechanism of HLJDD in AD was proposed, as shown in Figure 12.

Pruritus is a major clinical feature of AD, and AD is considered a prototype of chronic pruritic dermatosis (Ständer and Steinhoff, 2010). The pathogenesis of AD mainly occurs as a result of immune system disorders and epidermal barrier dysfunction, which may be related to genetic, immune, and environmental changes (Ständer and Steinhoff, 2010). Skin barrier damage and immunological dysfunction are key pathogenic components of AD. Th2 cells secrete cytokines, such as IL-4, IL-5, and IL-13, which are important for AD development, especially in the acute phase of AD. Skin barrier damage induces the release of large amounts of keratinocyte-derived proinflammatory cytokines, such as IL-33 and TSLP, from keratinocytes. It also activates the inflammatory cascade, mainly involving Th2 cytokines, such as IL-4, IL-13, and IL-5 (Weidinger et al., 2018). The results of our study indicated that the levels of IL-33, TSLP, IL-4, IL-13, and IL-5 were significantly upregulated in the AD model group, and that HLJDD and its fractions could reverse these effects. Histamine is the first and most well-known pruritogen (Simons and Simons, 2011). Itching sensation can be transmitted by the histamine pathway. In our study, the levels of histamine and IL-31 were significantly upregulated in the AD model group, and higher levels of IL-4Rα, JAK1, and HRH4 expression were detected in the AD model group by PCR and WB analysis. HLJDD and its fractions could also reverse these effects. Previous studies have indicated that IL-31, IL-4Rα, JAK1, and HRH4 are strongly associated with pruritus in AD (Oetjen et al., 2017). Therefore, HLJDD exhibited anti-AD effects by regulating IL-4Rα, JAK1, HRH4, histamine, and relative cytokines (IL-33, TSLP, IL-4, IL-13, and IL-5). 40AEF and CPF had much greater therapeutic action, which was considered the most important component of HLJDD for ameliorating AD.

Diseases often impair the normal metabolism of the body, and disordered metabolic conditions can be effectively measured by metabolomics studies, aiding in the diagnosis and treatment of diseases (Yu et al., 2021). Therefore, in the current study, we propose to use the untargeted and targeted metabolomics method to systematically explore the therapeutic mechanism of HLJDD and its fractions in the treatment of AD, which will allow us to specifically understand how the signaling has been altered during the treatment, and how changes in signaling can attenuate metabolic disorders in the body. Forty significant biomarkers were identified and selected using untargeted metabolomics. Metabolic pathway analysis indicated that these differential metabolites may be involved in histidine metabolism, arginine biosynthesis, pyrimidine metabolism, and alanine, aspartic acid, and glutamate metabolism, have been suggested to be subject to perturbation by HLJDD to improve the endogenous metabolism of AD. Further, for screening diagnostic biomarkers, Pearson’s correlation analysis was used for the correlation analysis of 17 physiological and biochemical indices and 40 potential biomarker data. Fourteen endogenous metabolites (DL-malic acid, glutaric acid, N-methyl-D-aspartic acid, linolelaidic acid, gamma-linolenic acid, L-alloisoleucine, L-carnosine, phosphocreatinine, L-alanine, 2′-deoxyuridine, prostaglandin D1, 1H-pyrimidine-2,4-dione, L-alanyl-L-glutamine, and biliverdine) were selected for targeted metabolomic analysis. The results of targeted metabolomics analysis indicated that these metabolites were restored to varying degrees in the HLJDD group, which was also consistent with the findings of untargeted metabolomics analysis. Meanwhile, we found that these metabolites were also restored to varying degrees in the CPF, 40AEF, 90AEF, WEF, and PEF groups, especially HLJDD, 40AEF, and CPF. Interestingly, 40AEF and CPF had much greater therapeutic action, which further validated that 40AEF and CPF were the most important components of HLJDD for ameliorating AD. In addition, the results of univariate and multivariate ROC curve-based exploratory analysis indicated that these metabolites may be potential candidate biomarkers for predicting AD.

Among these differential metabolites, fatty acids, including gamma-linolenic acid and linolelaidic acid, were increased in the AD model group. Fatty acids have been considered to play a critical role in inflammatory responses because they are a source of various lipid mediators (Sala-Vila et al., 2010; Schafer and Kragballe, 1991; Trak-Fellermeier et al., 2004). Gamma-linolenic acid has been reported to reduce skin water loss and promote skin epidermal cell proliferation. In patients with dry skin and moderate AD, long-term diet treatment with high gamma-linolenic acid has been found to gradually restore skin barrier function, reduce water loss in the skin, and decrease inflammatory factor expression in AD patients (Kawamura et al., 2011). Previous studies have revealed that prostaglandin D1 upregulation is involved in the prevention of AD by suppressing both mast cell and keratinocyte activation, which is one mechanism by which dihomo-g-linolenic acid prevents the development of atopic dermatitis (Amagai et al., 2015). Interestingly, higher levels of gamma-linolenic acid, linolelaidic acid, and prostaglandin D1 in the AD model group were observed in our study. After treatment, the levels of gamma-linolenic acid, linolelaidic acid, and prostaglandin D1 were downregulated in HLJDD and its fractions. Therefore, in the event of AD, there was a need for more gamma-linolenic acid, linolelaidic acid, and prostaglandin D1 in order to combat mast cell and keratinocyte activation. After treatment, higher levels of metabolites were also improved over time following the symptoms of AD amelioration. Thus, HLJDD may play an anti-inflammatory role during the treatment of AD by downregulating the expression of gamma-linolenic acid. The levels of amino acids, including L-alloisoleucine, N-methyl-D-aspartic acid, and L-alanyl-L-glutamine, were decreased and L-alanine was increased in the AD model group. Specifically, isoleucine is a branched-chain amino acid (BCAA). Changes in the metabolites involved in the BCAA pathway are known to influence the regulation of mTOR (Jewell and Guan, 2013; Zhenyukh et al., 2017), which plays a vital role in epidermal barrier formation and the signaling axis for the control of filaggrin as the fundamental pathophysiology of AD (Xda et al., 2020; Yang et al., 2014). Both N-methyl-D-aspartate and L-alanyl-L-glutamine are important excitatory neurotransmitters in the central nervous system and are associated with scratching and pathological modification behavior in AD-like mice (Fujii et al., 2018; Funakushi et al., 2011). The etiology of AD has been linked to deficiencies in the histamine-rich epidermal barrier protein filaggrin (Palmer et al., 2006). Carnosine and histamine are involved in histidine metabolism; their levels were significantly upregulated in the AD model group, demonstrating that histidine metabolism disorders occurred in AD. Similarly, the levels of these amino acids were reversed in the HLJDD and its fraction groups. The tricarboxylic acid (TCA) cycle, reflected in the altered DL-malic acid level found in this study, can shape immune cell responses via changes in the metabolic pathways of immune cells (O’Neill et al., 2016). In addition, the other differential metabolites (phosphocreatinine, 2′-deoxyuridine, 1H-pyrimidine-2,4-dione, and biliverdine) identified in this study also have implications for AD treatment.

## Conclusion

The present study utilized chemistry, biochemistry, and metabolomics analysis to dissect the pharmacological mechanism and substance basis of HLJDD in AD. This paper is the first to report on the development and validation of a multivariate analysis method to qualitatively and quantitatively analyze the non-similarity degree, which was suited to the non-similarity degrees of splitting fractions of HLJDD. Further, HLJDD exhibited anti-AD effects by inhibiting itching and enhancing immunity, which in turn regulating the levels of relative metabolites, and CPF and 40AEF were considered the most important components of HLJDD. This study provided novel insight into how chemistry, biochemistry, and metabolomics could be used as effective tools for elucidating the efficacy of HLJDD on AD.

## Data Availability

The original contributions presented in the study are included in the article/Supplementary Material, further inquiries can be directed to the corresponding author.
