# Supplementary material for: Study on the Molecular Basis of Huanglian Jiedu Decoction Against Atopic Dermatitis Integrating Chemistry, Biochemistry, and Metabolomics Strategies
Source: Front Pharmacol. 2021 Dec 14;12:770524. doi: 10.3389/fphar.2021.770524 (PMC8712871; doi:10.3389/fphar.2021.770524)
Supplement: Supplementary file 1 [file DataSheet1.ZIP › Supplemental Material/Fig. S1-S4.docx]

**Fig. 1 The HPLC fingerprints of PEF**


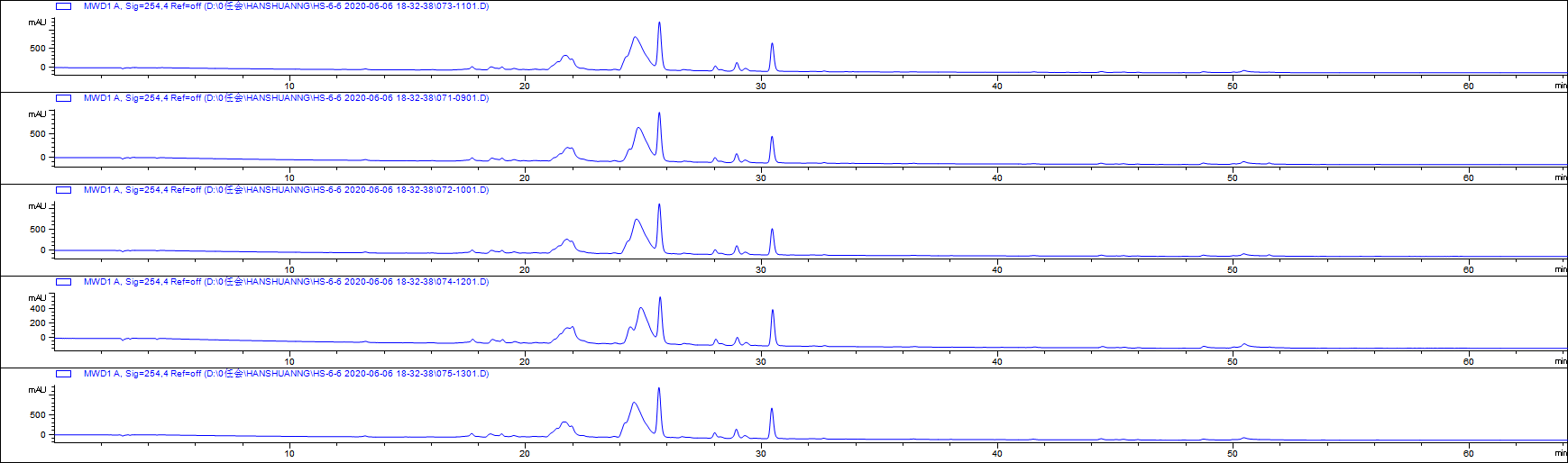


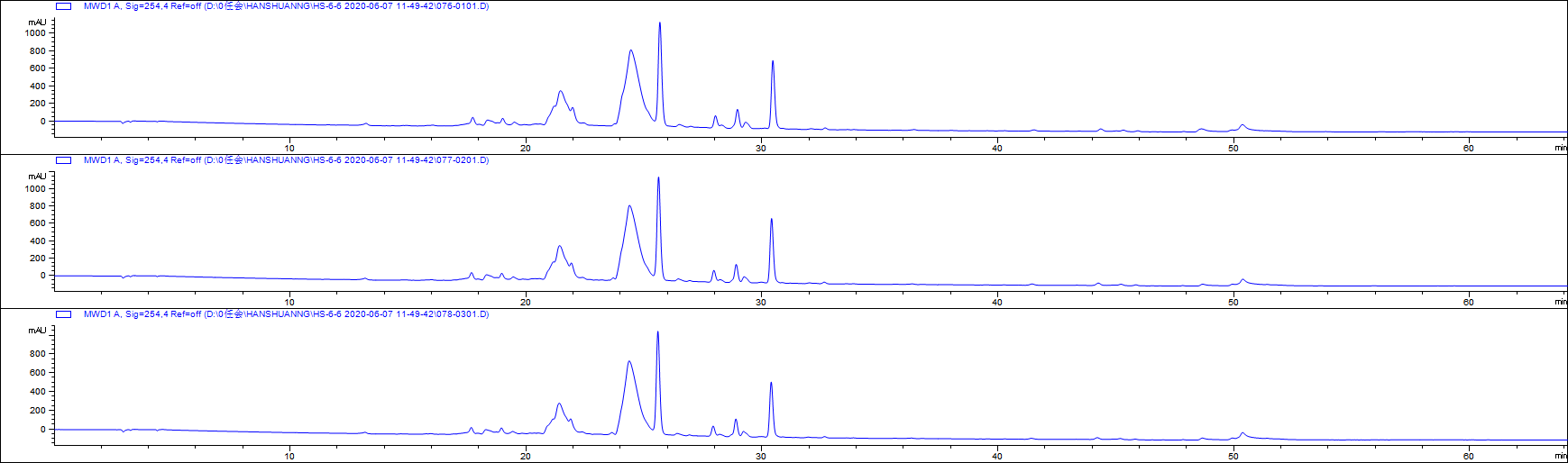


**Fig. 2 The HPLC fingerprints of 90AEF**


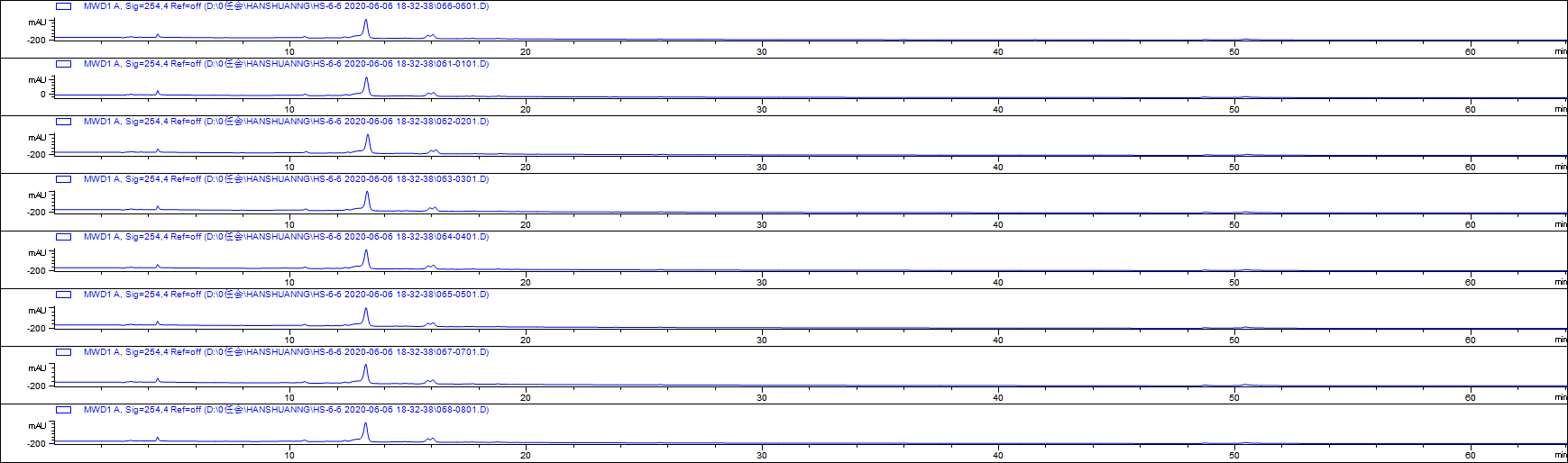


**Fig. 3 The HPLC fingerprints of 40AEF**

**Fig. 4 The HPLC fingerprints of WEF**
