# Supplementary material for: Study on the Molecular Basis of Huanglian Jiedu Decoction Against Atopic Dermatitis Integrating Chemistry, Biochemistry, and Metabolomics Strategies
Source: Front Pharmacol. 2021 Dec 14;12:770524. doi: 10.3389/fphar.2021.770524 (PMC8712871; doi:10.3389/fphar.2021.770524)
Supplement: Supplementary file 1 [file DataSheet1.ZIP › Supplemental Material/Fig. S5-S8.docx]

**Fig. 5 Fragmentation behavior of Berberine in Positive ion MS**

**Fig. 6 The mass spectrometry cleavage pathway of baicalin in positive ion mode.**

**Fig. 7 The mass spectrometry cleavage pathway of geniposide in positive ion mode.**

**Fig. 8** The specific fragmentation process of 4-Ocaffeoylquinic acid in negative ion mode.
