# Supplementary material for: Study on the Molecular Basis of Huanglian Jiedu Decoction Against Atopic Dermatitis Integrating Chemistry, Biochemistry, and Metabolomics Strategies
Source: Front Pharmacol. 2021 Dec 14;12:770524. doi: 10.3389/fphar.2021.770524 (PMC8712871; doi:10.3389/fphar.2021.770524)
Supplement: Supplementary file 1 [file DataSheet1.ZIP › Supplemental Material/Fig. S9-S10.docx]

| 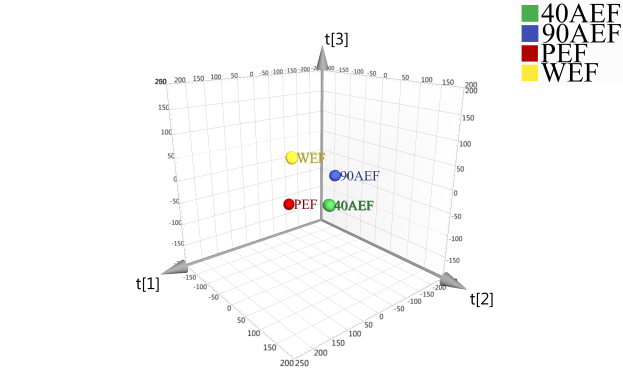 | 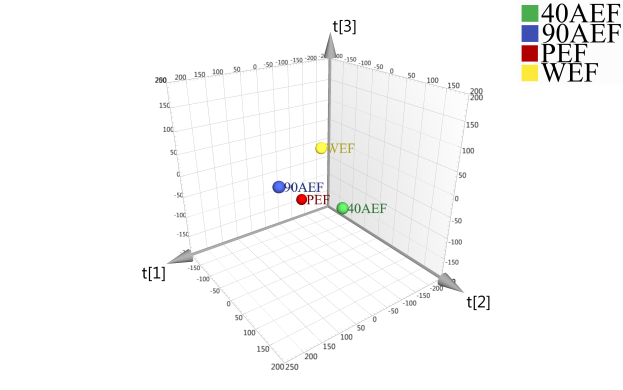 |
| --- | --- |
| **A** | **B** |
| **Fig. 9 The results of PCA in positive ion mode (A) and negative ion mode (B).** | |

**
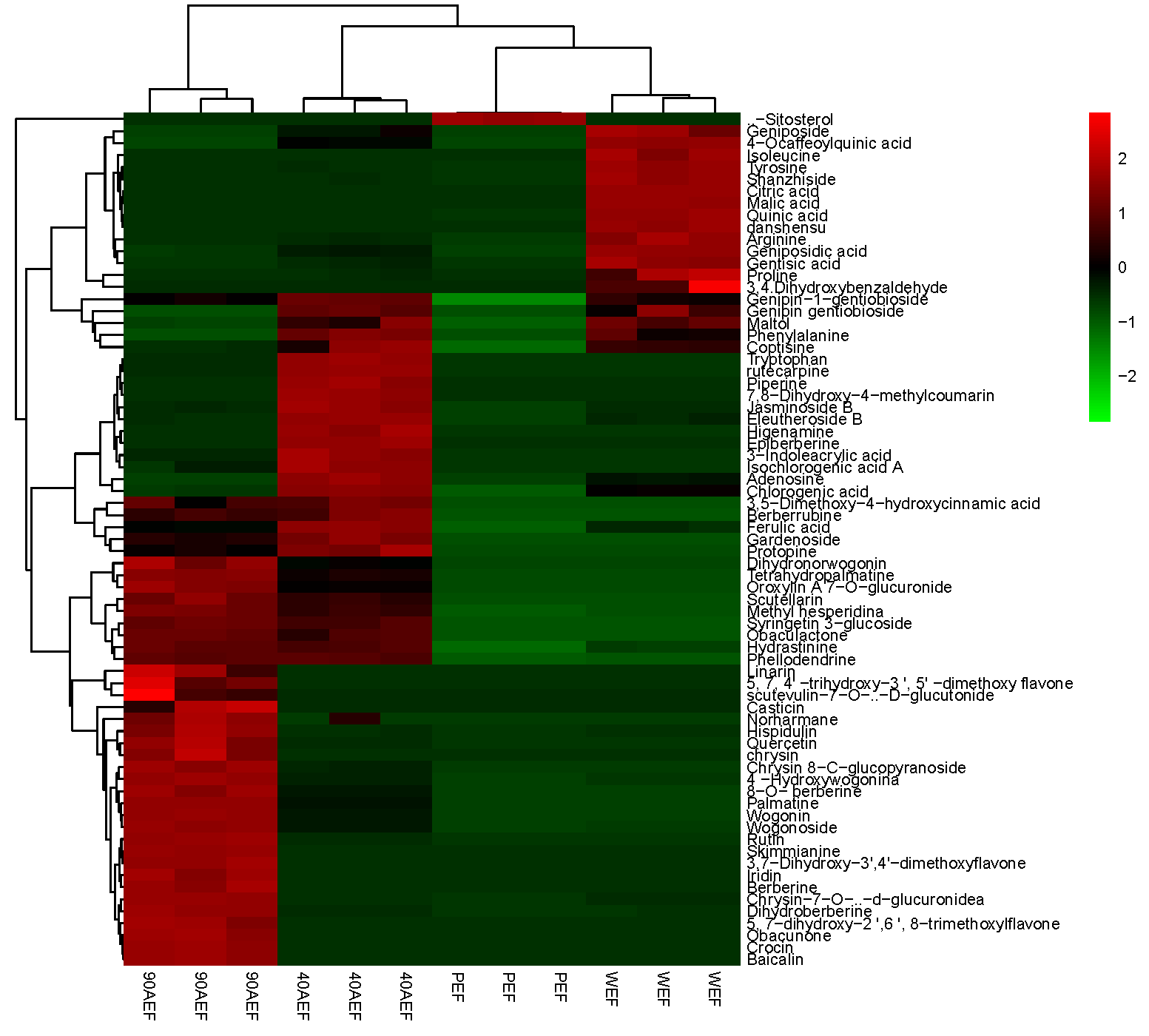
**

**Fig. 10 Hierarchical clustering analysis using data of 72 chemical components.**
