# Supplementary material for: Study on the Molecular Basis of Huanglian Jiedu Decoction Against Atopic Dermatitis Integrating Chemistry, Biochemistry, and Metabolomics Strategies
Source: Front Pharmacol. 2021 Dec 14;12:770524. doi: 10.3389/fphar.2021.770524 (PMC8712871; doi:10.3389/fphar.2021.770524)
Supplement: Supplementary file 1 [file DataSheet1.ZIP › Supplemental Material/Supplemental Material S1.docx]

Briefly, 1000 g of medicinal materials (four drugs including Coptidis Rhizoma, Phellodendri Chinensis Cortex, Scutellariae Radix and Gardeniae Fructus for ratio 3: 2: 2: 3) was pulverized and refluxed twice for 2 hour. Concentrate (0.3 g crude drug/mL) were processed by alcohol sedimentation (alcohol concentration were regulated as 75% using 95% ethanol). The precipitate and the supernatant were obtained. And the precipitate was washed four times by ethyl acetate to furnish the CPF. After removal of the alcohol, the supernatant were added water to concentration as 1 g crude drug/mL. Then, the supernatant was extracted three times with 60~90℃ petroleum ether to give PEF. Further, macroporous resin D101 was used to isolate the water layer and eluted with C_2_H_5_OH-H_2_0 (0, 40, 90% alcohol in water). The water eluates, 40% ethanol eluates and 90% ethanol eluates were collected, concentrated, lyophilized and evaporated in vacuum to give WEF, 40AEF and 90AEF. The whole process was repeated eight times and HLJDD were splitted as crude polysaccharides faction and four small molecular components.

The chromatographic column was Kromasil C18 (250 mm × 4.6 mm, 5 μm). The flow rate was set at 0.8 mL/min, the wavelength set at 254 nm and the temperature of the column was set at 25℃. The mobile phase was composed of water (A) and acetonitrile (B). The linear elution gradient program was used as follows: 0 min, 10%A; 0～41 min, 10%～50% A; 41～45 min, 50%～80% A; 45～55 min, 80%～90% A; 55～60 min, 90% A; 60～61 min, 90～10% A; 61～75 min,10% A. Sample injection volume was set at 10 𝜇L.
