# Supplementary material for: Study on the Molecular Basis of Huanglian Jiedu Decoction Against Atopic Dermatitis Integrating Chemistry, Biochemistry, and Metabolomics Strategies
Source: Front Pharmacol. 2021 Dec 14;12:770524. doi: 10.3389/fphar.2021.770524 (PMC8712871; doi:10.3389/fphar.2021.770524)
Supplement: Supplementary file 1 [file DataSheet1.ZIP › Supplemental Material/Supplemental Material S2.docx]

#### UPLC-Q-TOF/MS/MS conditions

A Shimadzu Nexera ultra-high performance liquid chromatography LC-30A system was used to UPLC-Q-TOF/MS/MS analysis. The column was a waters UPLC BEH C18 column (1.7 μm 2.1 * 100 mm). The flow rate was set at 0.4 mL/min and sample injection volume was set at 3 𝜇L. The mobile phase was composed of water (A) and 0.1% formic acid in acetonitrile (B). The linear elution gradient program was used as follows: 0-3.5 min, 95–85% A; 3.5-6 min, 85-70% A; 6-6.5, 70-70% A; 6.5-12 min, 70 -30% A; 12-12.5 min, 30-30% A; 12.5-18 min, 30-0 % A; 18-22 min, 0-0% A.

The MS analysis was carried out using an AB SCIEX Triple TOF 5600 mass spectrometer. During each acquisition cycle, the strongest molecular ions greater than 100 were screened and the corresponding MS/MS data were further acquired. ESI heater temperature was maintained at 550 ℃; pressures of nebulizer gas, auxiliary gas and curtain gas were set at 55 psi, 55 psi and 35 psi respectively. Collision energy and energy spread were set at 40 V and 20 V. Besides, Ion Spray Voltage Floating was set to 5500 V in positive ion mode and -4000 V in negative mode respectively.

#### Identification of chemical components

According to the chemical composition of each Chinese medicinal material, the peak alignment and peak matching were firstly carried out by Progenesis QI software (waters, America). The retention time, exact mass and chemical quantity of chemical components in HLJDD was obtained. Further, the chemical components in HLJDD were analyzed and identified by comparing the standard substance, literature and possible chemical classes and mass spectrometry cleavage rules.
