# Supplementary material for: Study on the Molecular Basis of Huanglian Jiedu Decoction Against Atopic Dermatitis Integrating Chemistry, Biochemistry, and Metabolomics Strategies
Source: Front Pharmacol. 2021 Dec 14;12:770524. doi: 10.3389/fphar.2021.770524 (PMC8712871; doi:10.3389/fphar.2021.770524)
Supplement: Supplementary file 1 [file DataSheet1.ZIP › Supplemental Material/Supplemental Material S3.docx]

The specific algorithm of three methods was as follows:

①Cross analysis method of peak areas of crude drug

$\left[ \begin{aligned} YA \\ \mathrm{YB} \\ \mathrm{YC} \\ \mathrm{YD} \end{aligned} \right]$ =$\left[ \begin{matrix} X1,1 & X1,2 & \ldots& X1,j \\ X2,1 & X2,2 & \ldots& X2,j \\ X3,1 & X3,2 & \ldots& X3,j \\ X4,1 & X4,2 & \ldots& X4,j \end{matrix} \right]$

a. X_1,j_ < X_2,j_, formula (1):

SD_1,2_ = $\sum_{f1}^{n} \left[ \begin{matrix} \frac{X1,j}{X2,j} & \frac{X1,j+X2,j}{\left( X1,1+X1,2+\ldots+X1,j+\ldots X1,n \right)+\left( X2,1+X2,2+\ldots+X2,j+\ldots X2,n \right)} \end{matrix} \right]$ (1)

b. X_1,j_ > X_2,j_, formula (2):

SD_2,1_ = $\sum_{f1}^{n} \left[ \begin{matrix} \frac{X2,j}{X1,j} & \frac{X1,j+X2,j}{\left( X1,1+X1,2+\ldots+X1,j+\ldots X1,n \right)+\left( X2,1+X2,2+\ldots+X2,j+\ldots X2,n \right)} \end{matrix} \right]$ (2)

Note: Y_A_, Y_B_, Y_C_ and Y_D_ represented the common peak areas of PEF, WEF, 40AEF and 90AEF. For example, calculation method for SD of PEF and WEF showed as formula (1) and formula (2).

② Angle cosin analysis method As far as this method is concerned, two fractions A and B were transformed into fuzzy weight vector and two fractions were distinguished accordance with qualitative and quantitative differences. The specific algorithm was as follows formula (3).

SD (Cosα) =$\frac{\sum_{i=1}^{n} a_{i}b_{i}}{\sqrt{\sum_{i=1}^{n} a_{i}^{2}\sum_{i=1}^{n} b_{i}^{2}}}$ (3)

Note: n was the number of common peaks of fractions A and B; a was peak area of i’th peak in fractions A; b was peak area of i’th peak in fractions B.

③ Correlation coefficient analysis method This method also was called pearson correlation coefficient method and was centralization of cosin. It was a shape measure and used to measure the correlation between variables. The specific algorithm was as follows formula (4).

SD=$\frac{\sum_{i}^{n} (a_{i}-\bar{a})(b_{i}-\bar{b})}{\sqrt{\sum_{i}^{n} \left( a_{i}-\bar{a} \right)^{2}\sum_{i}^{n} \left( b_{i}-\bar{b} \right)^{2}}}$ (4)

Note: n was the number of common peaks of fractions A and B; a was peak area of i’th peak in fractions A; b was peak area of i’th peak in fractions B.
