# Supplementary material for: Study on the Molecular Basis of Huanglian Jiedu Decoction Against Atopic Dermatitis Integrating Chemistry, Biochemistry, and Metabolomics Strategies
Source: Front Pharmacol. 2021 Dec 14;12:770524. doi: 10.3389/fphar.2021.770524 (PMC8712871; doi:10.3389/fphar.2021.770524)
Supplement: Supplementary file 1 [file DataSheet1.ZIP › Supplemental Material/Supplemental Material S4.docx]

A DNFB-induced AD model was established and used to explore the effects of HLJDD (3.2 g/kg, 6.4 g/kg, and 12.8 g/kg). Evaluation of dorsal skin lesions and ear swelling were shown in **Figure 1**. Further, cytokines (histamine, IgE, IL-3, IL-4, IL-5, IL-13, IL-17A, IL-31, IL-33), gastrin-releasing peptide (GRP), substance P (SP), and transient receptor potential cation channel subfamily V member 1 (TRPV-1) in the serum were detected. And the results were described as in **Figure 2.** The above results indicate that 12.8 g/kg was found to be the effective dose.


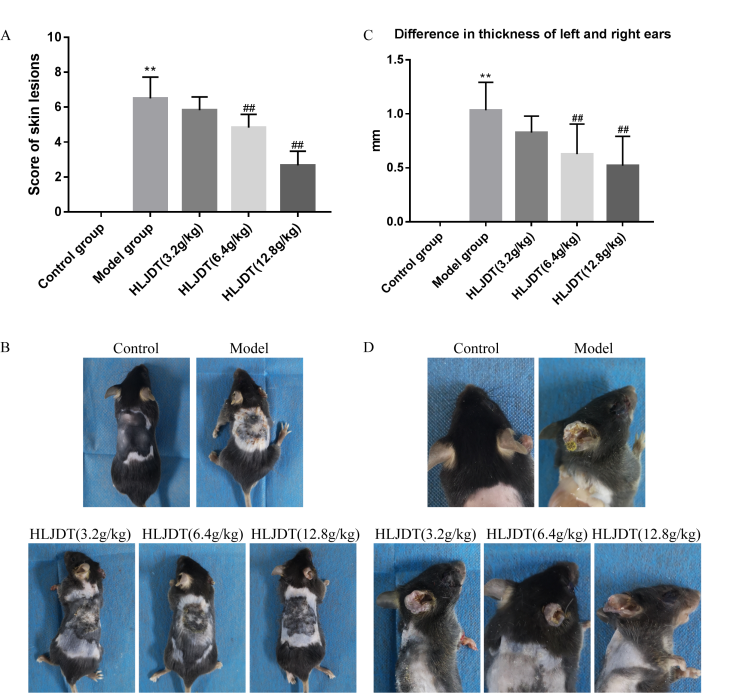


Figure 1 Effects of HLJDD (3.2 g/kg, 6.4 g/kg, and 12.8 g/kg) on dorsal skin lesions and ear swelling in AD.


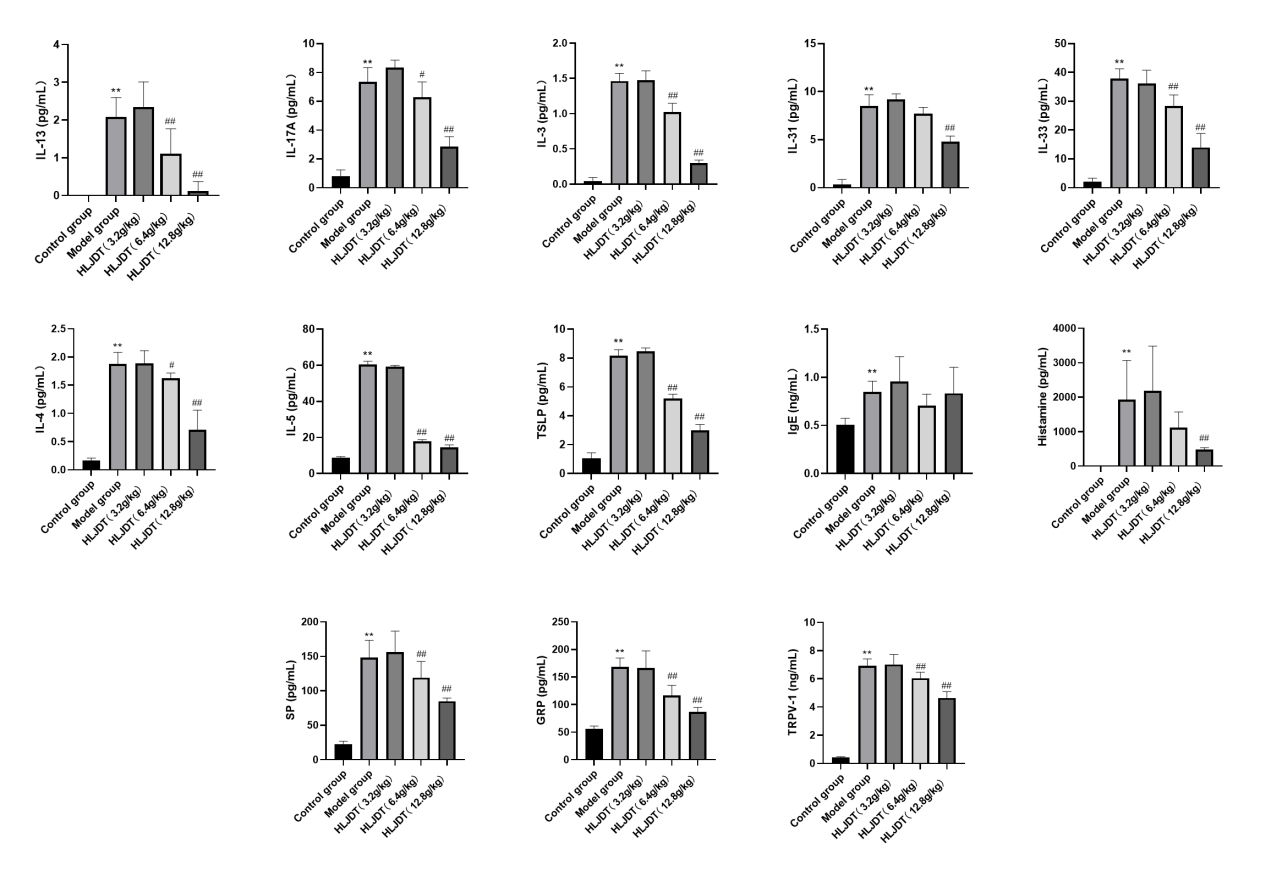


Figure 2 Effects of HLJDD (3.2 g/kg, 6.4 g/kg, and 12.8 g/kg) on cytokines (histamine, IgE, IL-3, IL-4, IL-5, IL-13, IL-17A, IL-31, IL-33), GRP, SP, and TRPV-1 in AD.
