# Supplementary material for: Study on the Molecular Basis of Huanglian Jiedu Decoction Against Atopic Dermatitis Integrating Chemistry, Biochemistry, and Metabolomics Strategies
Source: Front Pharmacol. 2021 Dec 14;12:770524. doi: 10.3389/fphar.2021.770524 (PMC8712871; doi:10.3389/fphar.2021.770524)
Supplement: Supplementary file 1 [file DataSheet1.ZIP › Supplemental Material/Supplemental Material S5.docx]

#### UPLC-Q/TOF-MS Conditions

Serum (100 μL) was added into 400 μL extract solution (acetonitrile: methanol = 1:1) containing internal standard (Adonitol, 1 μg/mL), vortexed for 30s and sonicated for 5 min in ice-water bath. The extraction was incubated at -40℃ for 1 h and then centrifuged at 10000 rpm for 15 min at 4 ℃. Next, 400 μL of supernatant was collected and dried at 37℃ in a vacuum concentrator. The dried samples were reconstituted in 200 μL of 50% acetonitrile by sonication on ice for 10 min. After centrifugation for 15 min at 13000 rpm at 4℃, an aliquot of 75 μL was transferred to a fresh glass vial for LC/MS analysis. The quality control (QC) sample was prepared by mixing an equal aliquot of the supernatants from all the samples. Chromatographic separation was performed on a UPLC BEH amide column (2.1 × 100 mm, 1.7 μm, Waters) using ExionLC Infinity series UHPLC System (AB Sciex). Keeping the temperature of the column at 25℃, we eluted the solvent using mobile phase of (A):ammonium acetate and ammonia hydroxide water solution with a concentration of 25 mmol/L and pH 9.7, and (B): acetonitrile, separately. The elution conditions were as following: 0-0.5 min, 95% B; 0.5-7.0 min, 95% B to 65% B; 7.0~8.0 min, 65% B to 40% B; 8.0-9.0 min, 40% B; 9.0-9.1 min, 40% B to 95% B; 9.1-12.0 min, 95% B. In the positive and negative ion mode, the sample loading volume was 2μL.

Mass spectrometry was performed on the Triple TOF 5600 mass spectrometry (AB Sciex) with an acquisition software (Analyst TF 1.7, AB Sciex) using information-dependent acquisition (IDA) mode. Depending on the preselected criteria of MS data, the MS/MS spectra were collected and triggered. The most intensive 12 precursor ions with intensity above 100 were opted for MS/MS in each cycle (cycle time was 0.56 s) at collision energy (CE) of 30 eV. The conditions of ESI source were described as follows: gas 1, gas 2 and curtain gas were set as 60 psi, 30 psi and 35 psi, respectively; source temperature 600℃, declustering potential 60V and ion spray voltage floating (ISVF) 5000V (positive mode) or 4000V (negative mode).

MS raw data (.wiff) files were converted to the mzXML format by ProteoWizard, and processed (including peak deconvolution, alignment, and integration) using R package XCMS (version 3.2). Minfrac and cut off were set as 0.5 and 0.3 respectively. In-house MS2 database was applied for metabolites identification.

#### Data processing and analysis

The raw data of UPLC/Q-TOF-MS/MS were initially processed using MarkerLynx 4.1 software. Principal component analysis (PCA) and orthogonal partial least squares discrimination analysis (PLS-DA) analysis were performed using SIMCA 14.1 software package (Sartorius Stedim Data Analytics AB, Umea, Sweden). The metabolites with VIP >1 and p < 0.05 were used as candidate metabolites. Metabolite Identification and Metabolic Annotation Candidate metabolites were identified by comparing their retention times and mass spectra with those of the standards and the mass spectra data at METLIN database (<http://metlin.scripps.edu/>), HMDB database (<http://www.hmdb.ca/>), and KEGG database (<http://www.genome.jp/kegg/>). Pathway analysis was performed with the MetPA database (<http://metpa.metabolomics.ca./MetPA/faces/Home.jsp>).
