# Supplementary material for: Study on the Molecular Basis of Huanglian Jiedu Decoction Against Atopic Dermatitis Integrating Chemistry, Biochemistry, and Metabolomics Strategies
Source: Front Pharmacol. 2021 Dec 14;12:770524. doi: 10.3389/fphar.2021.770524 (PMC8712871; doi:10.3389/fphar.2021.770524)
Supplement: Supplementary file 1 [file DataSheet1.ZIP › Supplemental Material/Supplemental Material S6.docx]

#### Targeted metabolomics sample processing and analyses

LC-MS/MS analysis was performed on a SCIEX ultra-high performance liquid chromatography LC-30A system with a Waters ACQUITY UPLCTM HSS C18 column (1.8 μm 2.1 * 100 mm). The flow rate was set at 0.4 mL/min and the sample injection volume was set at 1 μL. The mobile phase consisted of 0.1% formic acid in water (A) and 0.1% formic acid in acetonitrile (B). The multi-step linear elution gradient program was as follows: 0-2.5 min, 95–75% A; 2.5-3.5 min, 75-50% A; 3.5-4.5 min, 50-25% A; 4.5-7.5 min, 25-25% A; 7.5-8min, 25-0% A; 8-17min, 0-0% A.

An AB SCIEX Triple TOF 5600 mass spectrometer coupled with an Analyst TF 1.7.1 software was employed to obtain the MS and MS/MS data based on the IDA acquisition mode. During each acquisition cycle, the strongest molecular ions greater than 100 were screened and the corresponding MS/MS data were further acquired. ESI heater temperature was maintained at 550 ℃; pressures of nebulizer gas, auxiliary gas and curtain gas were set at 55 psi, 55 psi and 35 psi respectively. Collision energy and energy spread were set at 40 V and 20 V. Besides, Ion Spray Voltage Floating was set to 5500 V in positive ion mode and -4000 V in negative mode respectively.
