# Supplementary material for: Study on the Molecular Basis of Huanglian Jiedu Decoction Against Atopic Dermatitis Integrating Chemistry, Biochemistry, and Metabolomics Strategies
Source: Front Pharmacol. 2021 Dec 14;12:770524. doi: 10.3389/fphar.2021.770524 (PMC8712871; doi:10.3389/fphar.2021.770524)
Supplement: Supplementary file 1 [file DataSheet1.ZIP › Supplemental Material/Table S1.docx]

Table 1 MRM parameters of 14 standard substances

| CAS | Name | Parent ion | Daughter ion | Ionspray voltage (V) | Ion source temperature (℃) | Cluster voltage (v) | Collision energy (v) |
| --- | --- | --- | --- | --- | --- | --- | --- |
| 617-48-1 | DL-Malic acid | 135 | 77.1 | 5500 | 550 ℃ | 200.3 | 29.9 |
| 110-94-1 | Glutaric acid | 133 | 87 | 5500 | 550 ℃ | 39.6 | 14.4 |
| 6384-92-5 | NMDA | 148.1 | 88 | 5500 | 550 ℃ | 53.7 | 16.8 |
| 506-21-8 | Linoelaidic acid | 281.2 | 81.1 | 5500 | 550 ℃ | 154.6 | 25.2 |
| 506-26-3 | γ-LINOLENIC ACID | 279.2 | 81.1 | 5500 | 550 ℃ | 40.4 | 24.2 |
| 1509-34-8 | L-Alloisoleucine | 132.1 | 86.1 | 5500 | 550 ℃ | 25 | 14.4 |
| 305-84-0 | L-Carnosine | 227.1 | 110.1 | 5500 | 550 ℃ | 84.8 | 30.2 |
| 5786-71-0 | Fosfocreatinine | 194 | 114.1 | 5500 | 550 ℃ | 85.8 | 19 |
| 56-41-7 | L-Alanine | 90.1 | 44 | 5500 | 550 ℃ | 39.1 | 13.7 |
| 951-78-0 | 2'-Deoxyuridine | 229.1 | 113 | 5500 | 550 ℃ | 82.1 | 14.4 |
| 17968-82-0 | Prostaglandin D1 | 377.2 | 359.2 | 5500 | 550 ℃ | 99.8 | 23.4 |
| 66-22-8 | Uracil | 113 | 70 | 5500 | 550 ℃ | 106.8 | 21.4 |
| 39537-23-0 | L-Alanyl-L-Glutamine | 218.1 | 84 | 5500 | 550 ℃ | 59.9 | 36.3 |
| 114-25-0 | Biliverdine | 583.3 | 297.1 | 5500 | 550 ℃ | 204.8 | 40 |
