# Supplementary material for: Study on the Molecular Basis of Huanglian Jiedu Decoction Against Atopic Dermatitis Integrating Chemistry, Biochemistry, and Metabolomics Strategies
Source: Front Pharmacol. 2021 Dec 14;12:770524. doi: 10.3389/fphar.2021.770524 (PMC8712871; doi:10.3389/fphar.2021.770524)
Supplement: Supplementary file 1 [file DataSheet1.ZIP › Supplemental Material/Table S2-S3.docx]

**Table 2 NSD of chemical fractions of HLJDT using HPLC data**

| Splitted chemical fractions | NS (%) | | |
| --- | --- | --- | --- |
|  | Cross analysis method of peak areas of crude drug | Angle cosin analysis method | Correlation coefficient analysis method |
| water eluated and 40% alcohol eluated fractions | 96.27 | 97.9 | 98.84 |
| water eluated and 90% alcohol eluated fractions | 98.34 | 97.36 | 97.90 |
| water eluated and Petroleum ether fractions | 99.99 | 99.94 | 99.94 |
| 40% alcohol eluated and 90% alcohol eluated fractions | 95.85 | 99.82 | 99.28 |
| 40% alcohol eluated and Petroleum ether fractions | 99.99 | 99.61 | 99.61 |
| 90% alcohol eluated and Petroleum ether fractions | 99.97 | 99.48 | 99.49 |

**Table 3 NSD of chemical fractions of HLJDT using HPLC data**

| Splitted chemical fractions | NSD (%) | | |
| --- | --- | --- | --- |
|  | crude drug | Angle cosin analysis | Correlation coefficient analysis |
| water eluated and 40% alcohol eluated fractions | 99.36 | 98.44 | 91.26 |
| water eluated and 90% alcohol eluated fractions | 98.76 | 92.98 | 86.12 |
| water eluated and Petroleum ether fractions | 99.99 | 81.43 | 86.01 |
| 40% alcohol eluated and 90% alcohol eluated fractions | 97.20 | 85.05 | 85.64 |
| 40% alcohol eluated and Petroleum ether fractions | 99.99 | 81.00 | 84.06 |
| 90% alcohol eluated and Petroleum ether fractions | 99.99 | 83.69 | 83.10 |
